# Creation of Zinc (II)-Complexed Green Tea and Its Effects on Gut Microbiota by Daily Green Tea Consumption

**DOI:** 10.3390/molecules30153191

**Published:** 2025-07-30

**Authors:** Tsukasa Orita, Daichi Ijiri, De-Xing Hou, Kozue Sakao

**Affiliations:** 1 The United Graduate School of Agricultural Sciences, Kagoshima University, Kagoshima 890-0065, Japan; 2 Joint Faculty of Veterinary Medicine, Kagoshima University, Kagoshima 890-0065, Japan; 3 Faculty of Agriculture, Kagoshima University, Kagoshima 890-0065, Japan

**Keywords:** green tea, EGCg, Zinc (II), Western diet, gut microbiota, dysbiosis

## Abstract

Although Zn (II)-(−)-Epigallocatechin gallate (EGCg) complex (Zn-EGCg) is known for its promising bioactivities, little attention has been paid to its incorporation into daily green tea consumption. In this study, we aimed to incorporate Zn (II) into green tea extract to promote the formation of Zn-EGCg complex within the tea matrix. We then investigated how the formation of Zn-complexed green tea extract (Zn-GTE) influences the gut microbiota in a Western diet (WD)-fed mouse model. Structural analyses using ultraviolet–visible spectroscopy (UV–Vis), Fourier-transform infrared spectroscopy (FT-IR), proton nuclear magnetic resonance (^1^H NMR), and powder X-ray diffraction (PXRD) suggested that Zn (II) interacted with hydroxyl groups of polyphenols within the extract, consistent with Zn-EGCg formation, although the complex could not be unequivocally identified. Under intake levels equivalent to daily consumption, Zn-GTE administration restored WD-induced reductions in alpha-diversity and resulted in a distinct microbial composition compared to treatment with green tea extract (GTE) or Zn alone, as shown by beta-diversity analysis. Linear discriminant analysis Effect Size (LEfSe) analysis revealed increased abundances of bacterial taxa belonging to *o_Clostridiales*, *o_Bacteroidales*, and *f_Rikenellaceae*, and decreased abundances of *g_Akkermansia* in the Zn-GTE group compared to the GTE group. These findings highlight that Zn-GTE, prepared via Zn (II) supplementation to green tea, may exert distinct microbiota-modulating effects compared to its individual components. This study provides new insights into the role of dietary metal–polyphenol complexes, offering a food-based platform for studying metal–polyphenol interactions under physiologically relevant conditions.

## 1. Introduction

Green tea, a non-fermented tea, is derived from fresh leaves of the perennial plant *Camellia sinensis* (L.) O. Kuntze, and has attracted considerable global attention because of its rich polyphenols (20–30% of dry leaves) [1]. Various research has reported a broad spectrum of health-promoting properties of green tea, such as anti-obesity, anti-cancer, anti-allergy, and neuroprotective effects [2,3,4,5]. (−)-Epigallocatechin gallate (EGCg), the most abundant polyphenol in green tea, is a major contributor to the health-promoting effects of green tea [6]. However, several human randomized clinical trials have indicated that a relatively high daily dose of EGCg (135–856.8 mg EGCg/day) is required to achieve the health benefits of green tea [7]. This limitation is primarily attributed to the poor stability, absorption rate, and bioactivity of EGCg.

In recent years, many researchers have developed metal–EGCg complexes to overcome the limitations associated with EGCg, including its poor stability and low bioactivity. EGCg serves as an excellent metal ion chelator due to the presence of multiple phenolic hydroxyl groups in its molecular structure. Manganese (Mn), iron (Fe), copper (Cu), zinc (Zn), cadmium (Cd), and other metals have been used to form promising metal–EGCg complexes [8,9,10,11,12,13]. Among these, Zn plays a crucial role in human growth and development and is considered an ideal candidate for complex formation due to its strong chelating ability and excellent biocompatibility. It has been reported that EGCg can form a complex with Zn (II) and facilitate Zn (II) ion transport into cells by acting as an ionophore [14,15]. Moreover, the Zn-EGCg complex exhibits enhanced stability, antioxidant capacity, and anti-cancer activity compared to native EGCg [16,17].

Building on the accumulated knowledge of the Zn-EGCg complex, a wide range of possible applications has been proposed. Nevertheless, only a limited number of studies have examined the incorporation of the Zn-EGCg complex into green tea, a major dietary source of catechins, for practical use as a beverage. Zn-EGCg has been applied in delivery systems such as β-chitosan carriers for food or nutraceutical purposes [18]. In addition, ZnO/catechin nanocomposites have been synthesized using green tea extracts [19]. However, these approaches do not aim to facilitate the direct intake of Zn-EGCg as a natural component of green tea beverages. To the best of our knowledge, no study has demonstrated in situ formation of Zn-EGCg within a green tea infusion for dietary consumption. Pohl et al. reported that minerals such as Zn (II) exist in tea infusions bound to water-soluble polyphenols, such as catechins [20]. However, due to the complex organic composition of tea, direct structural evidence supporting the formation of Zn-EGCg in tea extracts remains scarce. Furthermore, the potential health benefits of green tea enriched with Zn-EGCg have yet to be thoroughly investigated.

Only 0.1–1.1% of orally ingested catechins reached systemic circulation, while approximately 69% were recovered in the ileal fluid as parent catechins and their metabolites [21,22]. Given this low absorption rate, it is widely accepted that the in vivo bioactivity of green tea is primarily mediated through action within the gastrointestinal tract. Notably, the anti-obesity effect of green tea is markedly attenuated in models with depleted intestinal microbiota, indicating that the gut microbiota plays a crucial role in mediating the effect of green tea [23]. Consequently, increasing attention has been directed toward the gut microbiota-modulating properties of green tea and its main catechin, EGCg. In human studies, green tea consumption has been shown to enhance the alpha-diversity at both genus and species levels in fecal samples [24]. Moreover, EGCg significantly increased the relative abundance of *Akkermansia muciniphila*, a beneficial bacterium known for its diabetes-ameliorating effects, in mice fed a high-fat diet (HFD) [25]. Although the impact of green tea on the gut microbiota has been extensively studied, to the best of our knowledge, there is currently no available research on the gut microbiota-modulating properties of the Zn-EGCg complex.

Alterations in the gut microbial community induced by the modern Western diet (WD), characterized by high intake of fat and refined sugars, known as dysbiosis, are associated with various host pathologies such as metabolic dysfunction-associated steatotic liver disease (MASLD), hypertension, and others [26,27,28]. Notably, WD intervention fails to induce obesity in germ-free mice, underscoring the pivotal role of dysbiosis in the development of host metabolic disorders [29]. Typical characteristics of dysbiosis include reduced microbial diversity, a decline in beneficial bacteria, and the overgrowth of pathogenic species [30,31]. Reduced gut bacterial richness and diversity have been widely implicated in the progression of diseases such as obesity [32]. Furthermore, WD-induced dysbiosis alters the gut microbiota composition in ways that adversely affect host metabolic functions, most notably by increasing the relative abundance of *Enterobacteriaceae* and decreasing the abundance of *Akkermansia muciniphila* and *Lactobacillus* [33,34].

Based on the above background, the present study explores a novel approach to understanding how Zn (II) interacts with green tea polyphenols under realistic consumption conditions. Specifically, we investigated whether the addition of Zn (II) to green tea extract (GTE) results in the formation of Zn-EGCg or other polyphenol–metal complexes, and whether such coordination can be characterized structurally within the complex matrix of tea extract. Furthermore, we focused on examining how the formation of Zn-based complexes in GTE may influence the gut microbiota, a critical mediator of host health. To this end, we prepared a Zn (II)-fortified and complex green tea extract (Zn-GTE) and evaluated its impact on gut microbial composition and activity in comparison with conventional GTE, using concentrations that reflect typical dietary intake.

Zn-GTE was prepared by simply adding a Zn (II) solution to brewed green tea, followed by stirring. To confirm the presence of the Zn-EGCg complex in Zn-GTE, we characterized the structural features of the purified Zn-EGCg complex using Fourier-transform infrared spectroscopy (FT-IR), ultraviolet–visible spectroscopy (UV–Vis), ^1^H nuclear magnetic resonance (NMR), and powder X-ray diffraction (PXRD) analysis. These structural markers were then used to verify the formation of the complex within the green tea matrix. The gut microbiota-modulating properties of Zn-GTE were evaluated using a WD-induced obesity model mouse, in which Zn-GTE was administered in an amount equivalent to typical beverage consumption. The WD used is characterized by high fat, high cholesterol, and high sugar content. Our findings highlight a novel Zn (II) fortification approach that modifies the health-promoting properties of green tea and offers new insights into the application of metal–polyphenol complexes in the development of functional foods.

## 2. Results

### 2.1. Structural Characterization of Zn-GTE and Zn-EGCg

#### 2.1.1. Analysis of Zn-GTE

To evaluate the structural changes induced by Zn (II) incorporation into green tea extract (GTE), Zn-GTE was analyzed and compared with GTE using UV–visible spectroscopy (UV–Vis), Fourier-transform infrared spectroscopy (FT-IR), proton nuclear magnetic resonance (^1^H NMR), and powder X-ray diffraction (PXRD). Zn-GTE was prepared by simply adding a Zn (II) solution to brewed green tea, followed by stirring.

**Figure 1:**
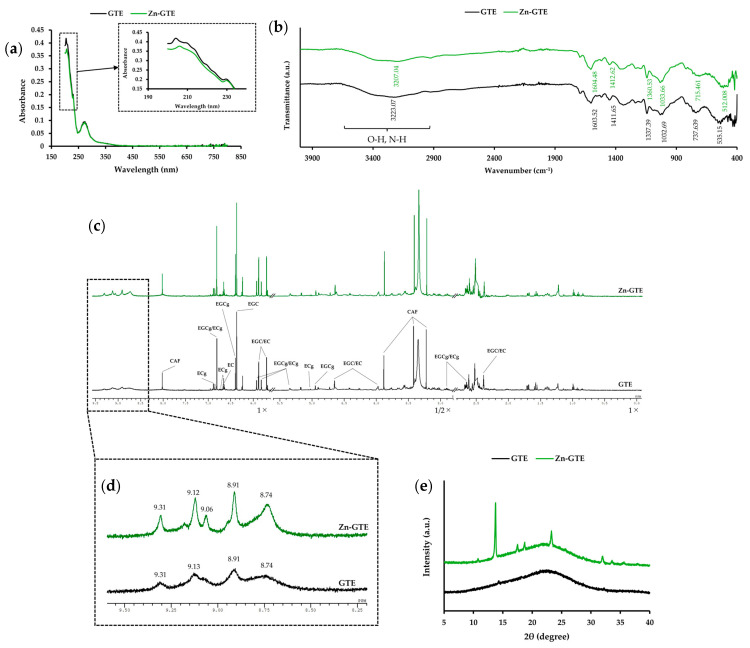
Structural characterization of Zn-GTE. (**a**) UV–Vis spectra of GTE and Zn-GTE in the wavelength range of 190–800 nm. (**b**) FT-IR spectra of GTE and Zn-GTE in the wavenumber range of 400–4000 cm^−1^. (**c**) Overview of the ^1^H-NMR spectra of GTE and Zn-GTE. (**d**) Enlarged spectral region (8.0–9.5 ppm) corresponding to the OH group of GTE and Zn-GTE. (**e**) PXRD patterns of GTE and Zn-GTE.

UV–Vis analysis was conducted to evaluate the light absorption characteristics of GTE and to estimate the presence of conjugated structures, which may be affected by Zn (II) complexation. UV–Vis spectroscopy revealed two absorption bands at 204 nm and 272 nm in GTE (Figure 1a). Upon Zn (II) addition, the 204 nm band slightly red-shifted to 206 nm with a decrease in intensity, while the 272 nm band remained unchanged. These spectral changes are consistent with interactions between Zn (II) and certain components of GTE, possibly polyphenols. However, direct evidence of complex formation was not obtained in this study.

To obtain information on functional groups in GTE, FT-IR analysis was conducted (Figure 1b). GTE showed a weak absorption peak at 3223.07 cm^−1^, attributable to the O-H and N-H stretching vibration. In Zn-GTE, this peak shifted to 3207.04 cm^−1^, indicating potential interactions between Zn (II) and hydroxyl or amide groups derived from polyphenols, polysaccharides, or proteins present in the extract. Additional peaks observed at 1337.39, 737.64, and 535.15 cm^−1^ in GTE were also shifted in Zn-GTE to 1360.53, 715.46, and 512.01 cm^−1^, respectively, supporting the presence of subtle structural changes. This result may support the hypothesis that Zn (II) interacts with functional groups of tea polyphenols, a key objective of this study.

^1^H NMR analysis was performed to detect changes in the proton environment by observing variations in chemical shifts, particularly in the aromatic hydroxyl region. As shown in Figure 1c, the ^1^H NMR spectra of both GTE and Zn-GTE exhibited multiple peaks derived from green tea metabolites, particularly catechins [35]. Since previous studies have revealed that metal ions could be coordinated to the hydroxy group of polyphenols [11], we focused on the aromatic hydroxy region (8.0–9.5 ppm) of the ^1^H NMR spectra (Figure 1d), which would allow us to detect potential changes due to metal coordination. GTE showed four broad peaks at 8.74, 8.91, 9.13, and 9.31 ppm, corresponding to protons of catechin’s hydroxyl group. Zn-GTE retained these signals but also exhibited a new, sharper peak at 9.06 ppm, indicating the possible presence of a more defined chemical environment, potentially resulting from coordination between Zn (II) and the hydroxyl groups of the catechins.

PXRD analysis was employed to assess crystallinity and structural organization in Zn-GTE, allowing us to detect any changes in crystal structure induced by Zn (II) addition. PXRD analysis (Figure 1e) showed no significant diffraction peaks for GTE, indicating an amorphous structure. In contrast, Zn-GTE displayed distinct peaks at 2θ values of 13.8°, 17.5°, 18.7°, 23.3°, and 32.0°, representing the formation of a partially crystalline structure, possibly due to coordination or aggregation involving Zn (II) and tea components.

Taken together, these results are consistent with Zn (II) interacting with polyphenolic and other constituents of green tea extract, leading to detectable structural changes. However, the formation of a defined Zn-EGCg complex remains to be confirmed through comparison with Zn-EGCg compounds formed using the standards.

#### 2.1.2. Analysis of Zn-EGCg

To determine whether the structural changes observed in Zn-GTE were attributable to Zn-EGCg complex formation, a synthesized Zn-EGCg complex was analyzed using UV–Vis, FT-IR, ^1^H NMR, and PXRD and compared with standard EGCg.

**Figure 2:**
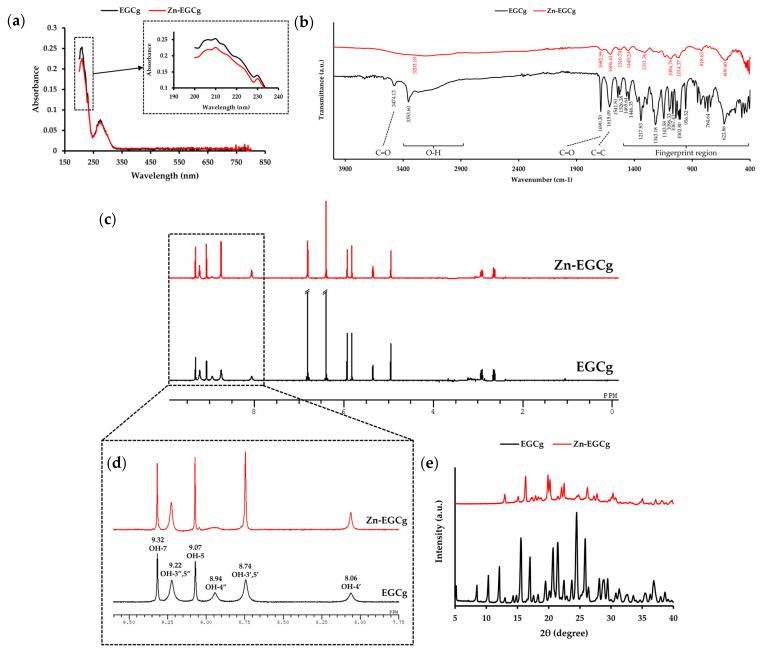
Structural characterization of Zn-EGCg. (**a**) UV–Vis spectra of EGCg and Zn-EGCg in the wavelength range of 190–800 nm. (**b**) FT-IR spectra of EGCg and Zn-EGCg in the wavenumber range of 400–4000 cm^−1^. (**c**) Overview of the ^1^H-NMR spectra of EGCg and Zn-EGCg. (**d**) Enlarged spectral region (8.0–9.5 ppm) corresponding to the OH group of EGCg and Zn-EGCg. (**e**) PXRD patterns of EGCg and Zn-EGCg.

In the UV–Vis spectra (Figure 2a), EGCg exhibited two characteristic absorption peaks at 210 nm and 274 nm. Upon complexation with Zn (II), the absorbance at 210 nm decreased, whereas the peak at 274 nm remained unchanged. The decrease in absorbance at 210 nm upon Zn (II) complexation mirrors the shift observed in Zn-GTE at 204 nm, suggesting that Zn-EGCg formation contributes to conjugation changes detectable within the green tea matrix.

FT-IR analysis revealed strong O–H stretching vibrations in EGCg at 3353.60 cm^−1^. In contrast, the Zn-EGCg complex showed an almost complete disappearance of the broad aromatic O–H bands, representing coordination between hydroxyl groups and Zn (II). The characteristic peak at 3473.13 cm^−1^ could be attributed to C=O stretching vibrational overtone. Moreover, a C=O stretching vibration at 1690.30 cm^−1^ and C=C stretching vibration at 1615.09 cm^−1^ were observed in EGCg. These peaks were altered in the Zn-EGCg complex. This change suggests that Zn (II) coordinates to the D ring of EGCg, affecting the C=O stretching vibration. Especially, the suppression of aromatic O–H vibrations in Zn-EGCg mirrors the spectral changes in Zn-GTE, partially supporting our hypothesis that Zn-EGCg complexation contributes to the structural alterations in the extract.

Figure 2c shows the ^1^H NMR spectrum of EGCg, which exhibits signals corresponding to eight hydroxyl and ten aromatic protons. This is consistent with previous reports [11]. In the aromatic O-H region (8.0–9.5 ppm, Figure 2d), the peak at 8.94 ppm, assigned to the 4”-OH, disappeared in Zn-EGCg. Additionally, the integral of the 8.74 ppm peak (3′,5′-OH) was reduced by nearly half compared to free EGCg, supporting coordination of the D-ring hydroxyl groups with Zn (II). On the other hand, the signals corresponding to other hydroxyl groups in Zn-EGCg became sharper compared to those observed in free EGCg. The sharpening of specific aromatic hydroxyl signals in Zn-EGCg replicates similar peak shifts seen in Zn-GTE, reinforcing the idea that Zn (II) selectively interacts with catechin hydroxyls both in isolated and extract contexts.

PXRD analysis (Figure 2e) showed that EGCg exhibited sharp crystalline peaks at 2θ values of 10.3°, 15.5°, 17.0°, 20.7°, 24.5°, and 25.9°, indicating its high crystallinity. In contrast, the Zn-EGCg complex displayed distinct diffraction peaks at 16.3°, 19.9°, 20.2°, 22.1°, 22.5°, and 26.2°, none of which overlapped with those of EGCg, suggesting the formation of a new crystalline phase distinct from that of the uncomplexed compound.

These spectral features observed for Zn-EGCg resembled some of the shifts detected in Zn-GTE. This raises the possibility that Zn-EGCg complex formation may contribute to the overall structural changes in Zn-GTE.

### 2.2. Catechin-Specific Contributions to Zn (II) Chelation in GTE

To elucidate the relative contribution of individual catechins to the Zn (II)-chelating capacity of GTE, a fluorescence-based assay was conducted using the Zn (II)-selective indicator FluoZin-3. Based on high performance liquid chromatography (HPLC) analysis, the concentrations of four major catechins in a 1.0 mg/mL GTE solution were estimated as follows: EGCg, 0.12 mg/mL; (−)-epicatechin gallate (ECg), 0.01 mg/mL; (−)-epigallocatechin (EGC), 0.10 mg/mL; and (−)-epicatechin (EC), 0.03 mg/mL (Table S1). Based on these concentrations, corresponding catechin standards were prepared to reflect their levels in GTE.

**Figure 3:**
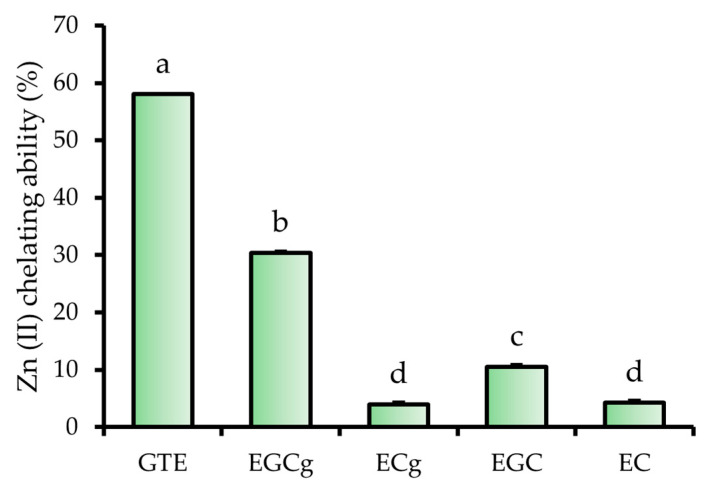
Zn (II) chelating ability of GTE, EGCg, ECg, EGC, and EC. The data represent mean ± SE, and different letters indicate significant differences (*p* < 0.05).

Zn (II) chelating ability was evaluated by measuring the quenching of FluoZin-3 fluorescence in the presence of 1 μM free Zn (II). The 1.0 mg/mL GTE sample quenched 58.0% of free Zn (II) (Figure 3). Under the same conditions, the Zn (II) chelating abilities of catechins were as follows: EGCg, 30.4%; ECg, 4.0%; EGC, 10.5%; and EC, 4.2%. EGCg exhibited the strongest Zn (II) chelating ability among the tested catechins, indicating that it was the predominant contributor to the overall Zn (II) chelating ability of GTE.

### 2.3. Effects of GTE and Its Zn (II) Complex on WD-Induced Gut Microbial Diversity

To investigate the modulatory effects of Zn-GTE on gut microbiota, we established a Western diet (WD)-induced dysbiosis model. Five-week-old mice were fed either a normal diet (ND) or WD for 12 weeks. GTE and Zn-GTE were administered to WD-fed mice at doses equivalent to typical beverage intake. A group receiving ZnSO_4_ 7H_2_O served as a Zn (II) control. At the end of the experiment, fecal samples were collected for 16S rRNA gene sequencing analysis.

**Figure 4:**
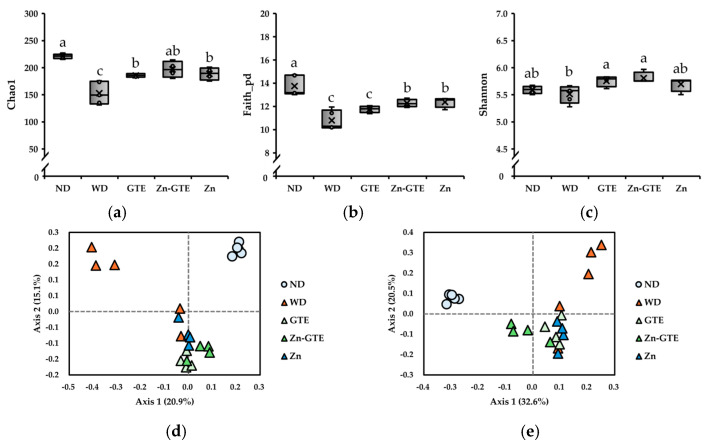
Effects of samples on diversity of mice gut microbiota. (**a**) Chao1 index. (**b**) Faith_pd index. (**c**) Shannon index. The data represent the median and range of values observed in mice. Different letters in the same column indicate significant differences (*p* < 0.05). Principal coordinate analysis (PCoA) plot based on (**d**) Jaccard distance and (**e**) Bray–Curtis distance.

We evaluated alpha-diversity using Chao1 (species richness), Faith’s phylogenetic diversity (Faith_pd, phylogenetic richness), and Shannon (species evenness) indices. As shown in Figure 4a,b, species richness (Chao1 and Faith_pd indices) significantly decreased in the WD group compared to the ND group, confirming that the WD induced gut dysbiosis. All treatments mitigated the WD-induced reduction in the Chao1 index. Notably, Zn-GTE and Zn supplementation significantly restored the Faith_pd index. In addition, both GTE and Zn-GTE improved species evenness, as indicated by the Shannon index (Figure 4c).

We also assessed beta-diversity (between-habitat diversity) to examine changes in the overall gut microbiota structure. Jaccard and Bray–Curtis distances were used to capture differences in taxonomic presence/absence and relative abundance, respectively. Pairwise permutational multivariate analysis of variance (PERMANOVA) analysis revealed significant differences between the ND and WD groups in both Jaccard (q = 0.030) and Bray–Curtis (q = 0.025) distance-based PCoA plots (Figure 4d,e). Interestingly, Bray–Curtis-based analysis showed that Zn-GTE induced a gut microbiota composition distinct from that of GTE (q = 0.038) and Zn (q = 0.038) (Figure 4e). In contrast, Jaccard-based comparisons between Zn-GTE and the other treatments were not statistically significant (GTE vs. Zn-GTE: q = 0.060; Zn vs. Zn-GTE: q = 0.055) (Figure 4d). These findings indicate that Zn-GTE modulated the relative abundance of certain taxa and established a microbial profile distinct from either GTE or Zn supplementation alone.

### 2.4. Pattern Differentiation of Gut Microbiota by GTE and Its Zn(II) Complex

**Figure 5:**
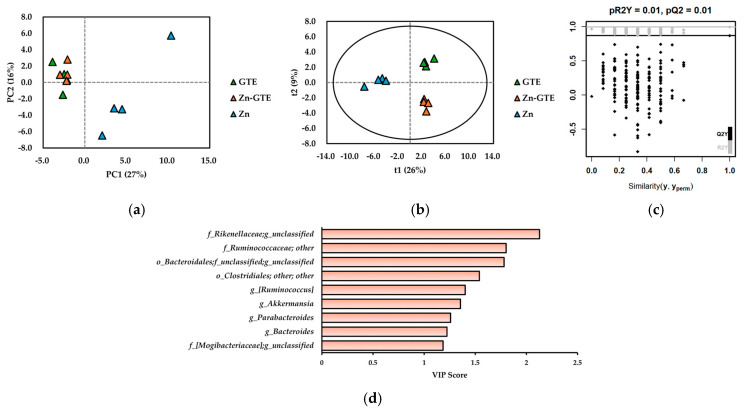
(**a**) Principal component analysis (PCA) score plot of gut microbiome data at genus level. (**b**) Projections to latent structures discriminant analysis (PLS-DA) score plot of gut microbiome data at genus level. (**c**) Validation plot obtained from 200-time permutation tests for PLS-DA model. (**d**) Variable importance in the projection (VIP) score obtained from PLS-DA model.

To identify key bacterial genera driving differences among the GTE, Zn-GTE, and Zn groups, we performed unsupervised principal component analysis (PCA) and supervised partial least squares discriminant analysis (PLS-DA) based on the relative abundances of 87 detected genera. These analyses were chosen to visualize and highlight the most influential genera contributing to the observed group differences and to determine the underlying microbial patterns associated with each treatment. As shown in Figure 5a, principal components 1 (PC1) and 2 (PC2) explained 27% and 16% of the total variance, respectively, and enabled classification of the treatment groups in the PCA score plot. The GTE and Zn-GTE groups clustered on the negative side of PC1, whereas the Zn group clustered on the positive side. However, GTE and Zn-GTE were not clearly separated in the PCA score plot.

To achieve better discrimination, we constructed a PLS-DA model. As illustrated in Figure 5b, the model clearly separated the three groups based on treatment (R^2^X = 0.569, R^2^Y = 0.994, Q^2^ = 0.867). A 200-time permutation test confirmed the validity of the model, as the permuted R^2^Y and Q^2^ values were substantially lower than those of the original model, indicating no overfitting (Figure 5c). To identify bacterial taxa contributing to group discrimination, we analyzed variable importance in projection (VIP) scores. Genera with VIP scores ≥1 were considered major contributors to classification. Figure 5d highlights bacterial genera with VIP scores ≥1 and relative abundances ≥1%. These taxes were mainly affiliated with the *p_Bacteroidetes* and *p_Firmicutes*, particularly the *o_Bacteroidales* (within *p_Bacteroidetes*) and *o_Clostridiales* (within *p_Firmicutes*). Among them, the *f_Rikenellaceae; g_unclassified* showed the highest VIP score, indicating its strong contribution to group discrimination. Due to taxonomic resolution limits of 16S rRNA sequencing, some genera remained unclassified. Notably, *g_Akkermansia*, a genus within the *p_Verrucomicrobia*, also exhibited a high VIP score.

### 2.5. Effects of GTE and Its Zn (II) Complex on WD-Induced Gut Microbial Composition

**Figure 6:**
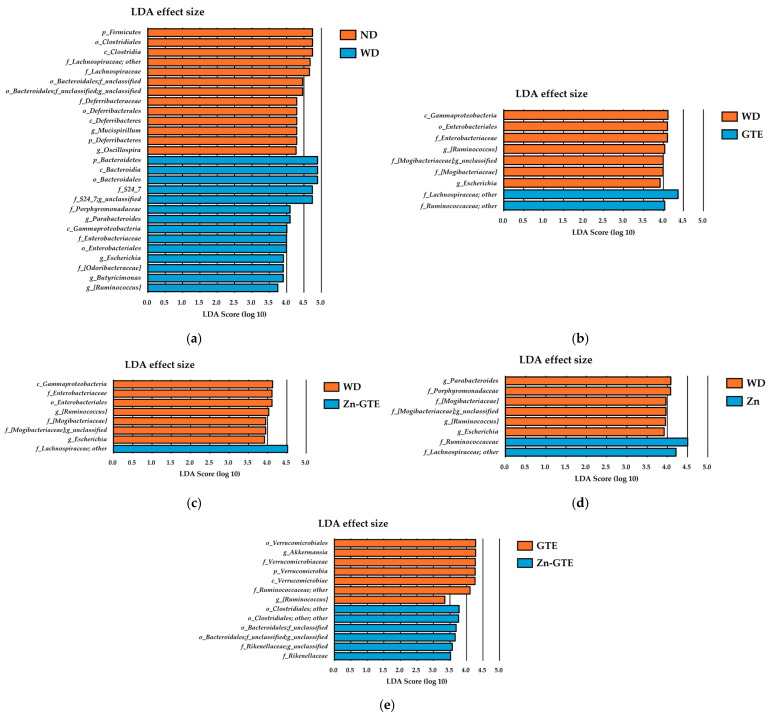
Effect of samples on abundant taxa of gut microbiota. (**a**) Differences in the composition of groups by linear discriminant analysis (LDA) effect size (LEfSe) analysis between ND and WD, (**b**) WD and GTE, (**c**) WD and Zn-GTE, (**d**) WD and Zn, and (**e**) GTE and Zn-GTE. The threshold for the logarithmic LDA score was set to 2.0.

To comprehensively identify candidate microbial biomarkers with significant differences in abundance across groups, we performed linear discriminant analysis effect size (LEfSe) analysis. This method was chosen to identify taxa with relative abundances ≥1% that most clearly differentiate between groups, providing insight into microbial features associated with the experimental conditions. Across taxonomic levels from phylum to genus, LEfSe identified 27 taxa between the ND and WD groups, 9 between WD and GTE, 8 between WD and Zn-GTE, 8 between WD and Zn, and 13 between the GTE and Zn-GTE groups, all with LDA scores ≥2.0 (Figure 6a–e).

In the GTE group compared with the WD group, several genera were significantly altered. Genera affiliated with *f_Lachnospiraceae* and *f_Ruminococcaceae* were enriched, while *g_[Ruminococcus]*, *f_[Mogibacteriaceae]*, and *g_Escherichia* were reduced (Figure 6b). Similarly, in the Zn-GTE group, only genera from the *f_Lachnospiraceae* showed increased abundance relative to the WD group, while the decreased taxa largely overlapped with those observed in the GTE group (Figure 6c). In the Zn group, both *f_Lachnospiraceae* and *f_Ruminococcaceae* families were enriched compared to WD-fed mice (Figure 6d).

Notably, distinct microbial biomarkers emerged in comparison between the GTE and Zn-GTE groups. The Zn-GTE group exhibited increased relative abundances of genera affiliated with *o_Clostridiales*, *f_Bacteroidales*, and *f_Rikenellaceae*, while *g_Akkermansia* and *g_[Ruminococcus]* were reduced (Figure 6e). These discriminatory taxa closely aligned with those identified by high VIP scores in the PLS-DA model, reinforcing their relevance. These findings suggest that complexation of GTE components with Zn (II) induces gut microbial composition changes distinct from those elicited by GTE alone.

### 2.6. Effects of GTE and Its Zn (II) Complex on WD-Induced Gut Microbial Metabolic Pathway

**Figure 7:**
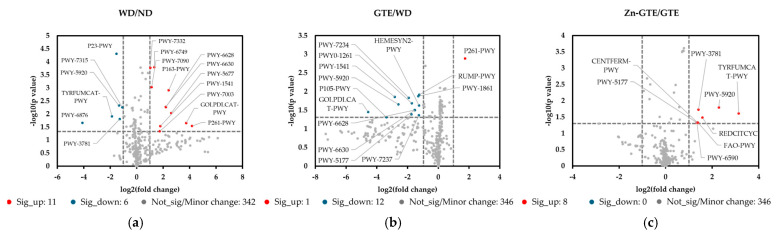
Effect of samples on gut microbial metabolic pathway by metagenome predictions (PICRUSt2) against Metabolic Pathways From all Domains of Life (MetaCyc) database. (**a**) Volcano plot based on the abundance of MetaCyc pathways between ND and WD, (**b**) WD and GTE, and (**c**) GTE and Zn-GTE.

Based on 16S rRNA gene sequencing data, we further predicted gut microbial metabolic pathways using the Metabolic Pathways From all Domains of Life (MetaCyc) database. To identify differences in the abundances of these pathways between groups, volcano plots were generated using a fold change (FC) threshold of 2 and a *t*-test significance level of 0.05 (Figure 7, Table S2).

In the WD group, 17 metabolic pathways exhibited significant differences compared to the ND group (Figure 7a). Specifically, the relative abundances of Gram-negative cell wall biosynthesis pathways (PWY-6749, PWY-7090, and PWY-7332), butyrate production pathways (PWY-5677 and P163-PWY), and amino acid biosynthesis pathways (PWY-6628 and PWY-6630) increased relative to the ND group.

In contrast, 12 pathways significantly decreased in the GTE group compared to the WD group. Among these, glycerol degradation (GOLPDLCAT-PWY), tricarboxylic acid (TCA) cycle IV (2-oxoglutarate decarboxylase) (P105-PWY), and taurine degradation (PWY-1541) showed the most pronounced reductions (Figure 7b).

In the Zn-GTE group, eight metabolic pathways, such as butyrate production (CENTFERM-PWY and PWY-6590), fatty acid β-oxidation (FAO-PWY), and TCA cycle (PWY-3781 and REDCITCYC) pathways, were significantly increased compared to the GTE group (Figure 7c). These findings highlight the distinct shifts in metabolic pathways induced by the complexation of Zn (II) with GTE, as well as its impact on gut microbial composition.

### 2.7. Effects of GTE and Its Zn (II) Complex on Organic Acid Composition in the Cecum

**Figure 8:**
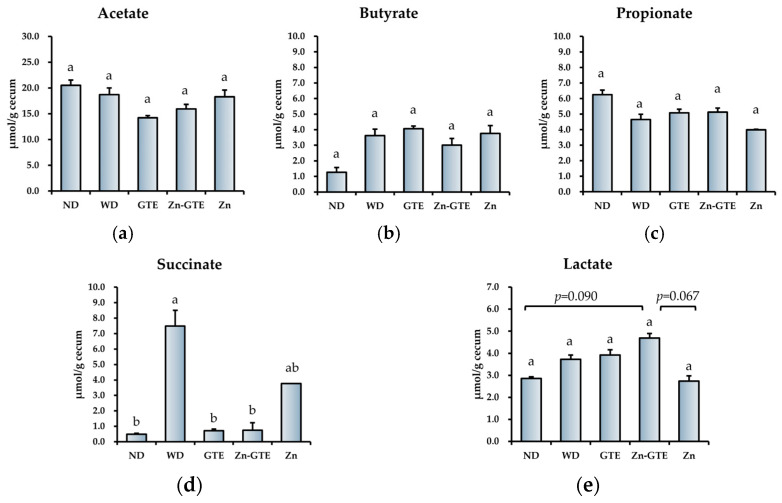
Effect of samples on organic acids’ composition in the cecum. (**a**) The content of acetate, (**b**) propionate, (**c**) butyrate, (**d**) succinate, and (**e**) lactate. The data represent mean ± SE, and different letters in the same column indicate significant differences (*p* < 0.05).

Since both pure GTE and Zn-GTE were each found to alter gut microbiota composition and related metabolic pathways, we measured the concentrations of organic acids in the cecum to evaluate functional metabolic changes. Among the major short-chain fatty acids—acetate, propionate, and butyrate—no significant differences were observed among the groups (Figure 8a–c). In contrast, succinate levels were significantly elevated in the WD group compared to the ND group, and this increase was markedly reversed by both GTE and Zn-GTE treatment (Figure 8d). Although lactate levels tended to increase in the Zn-GTE group compared to the other groups, the differences were not statistically significant (Figure 8e).

### 2.8. Effects of GTE and Its Zn (II) Complex on Serum and Hepatic Zn Status

**Figure 9:**
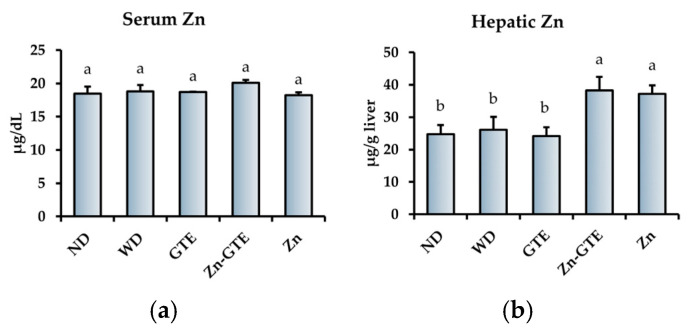
Effects of samples on Zn status. (**a**) Serum Zn content. (**b**) Hepatic Zn content. The data represent mean ± SE, and different letters in the same column indicate significant differences (*p* < 0.05).

We further evaluated the effects of the samples on Zn status in serum and liver. Figure 9 shows the serum and hepatic Zn contents across all experimental groups. No significant differences were observed in serum Zn levels among the groups (Figure 9a). However, hepatic Zn levels were significantly higher in the Zn-GTE and Zn groups compared to the WD group, whereas GTE alone had no significant effect relative to WD (Figure 9b).

## 3. Discussion

### 3.1. Chemical Structure of Zn-EGCg and Zn-GTE

The formation of Zn-EGCg complex was investigated to provide a structural model for understanding Zn (II) interactions in green tea extracts (Zn-GTE). The results of structural analyses demonstrated that Zn (II) forms complexes with EGCg, inducing distinct changes in its spectroscopic properties.

The UV–Vis spectral changes observed upon complexation of EGCg with Zn (II)—specifically, the decreased absorbance at 210 nm and the unchanged peak at 274 nm—can be rationalized by examining the underlying molecular interactions (Figure 2a). ^1^H NMR and FT-IR analyses indicated that Zn (II) preferentially coordinates to the phenolic hydroxyl groups of EGCg, particularly those located in the D ring (Figure 2b,c). This chelation restricts the delocalization of π electrons within the conjugated system, which is associated with the absorption near 210 nm. The diminished intensity at 210 nm is, therefore, best interpreted as an indirect perturbation of π → π* transitions. In contrast, the 274 nm peak remains unaffected by Zn (II) coordination, supporting the notion that chelation modulates electron delocalization only in specific regions of the molecule. These spectral observations are consistent with the structural data of Zn-GTE and help elucidate the selective modulation of the UV–Vis profile upon metal coordination.

**Figure 10:**
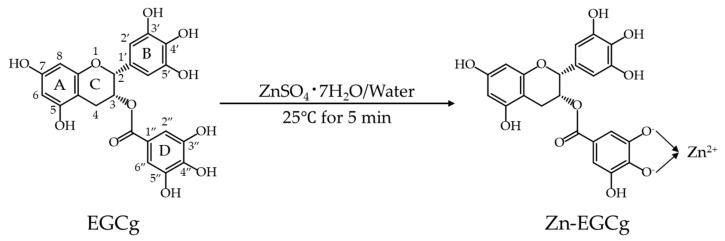
Presumed structure of Zn-EGCg in this study.

FT-IR analysis revealed a marked decrease or disappearance of the strong O-H stretching bands in Zn-EGCg compared to free EGCg (Figure 2b), indicating the involvement of phenolic hydroxyl groups in metal coordination. In particular, the ^1^H NMR analysis provided strong evidence for D-ring involvement, as the characteristic signals of hydroxyl protons (e.g., at 8.94 ppm) were either diminished or shifted (Figure 2d). This observation is consistent with previous studies suggesting preferential Zn (II) binding at the D-ring hydroxyls of EGCg [16], although other studies have reported B-ring involvement depending on reaction conditions [11]. Based on our data and prior findings, a tentative structure of Zn-EGCg is proposed (Figure 10), assuming a 1:1 molar coordination, which is known to be the most stable stoichiometry for Zn–catechin complexes [36].

To examine whether a similar Zn-EGCg complex is formed within the GTE matrix, spectroscopic comparisons were conducted between Zn-EGCg and Zn-GTE. A reduction in absorbance around 204 nm was observed in the UV–Vis spectra of Zn-GTE (Figure 1a), resembling the spectral changes seen in the Zn-EGCg complex. This suggests potential interactions between Zn (II) and polyphenolic components in the extract. However, in the UV region (190–500 nm) of tea, the observed transitions may include π → π* for compounds with conjugated double bonds, as well as n → σ* and n → π* transitions [37]. Thus, UV–Vis absorption in tea extracts could reflect the presence of other components, such as caffeine (absorbing around 275 nm) [38]. These spectral changes are not specific to EGCg. Therefore, UV–Vis analysis alone may not provide sufficient specificity to conclusively identify Zn–EGCg complexation within the GTE matrix. FT-IR analysis of Zn-GTE also showed shifts in the O-H and N-H stretching regions (Figure 1b), which may reflect coordination between Zn (II) and hydroxyl/amide groups derived from polyphenols, polysaccharides, or proteins [39]. However, due to the complex nature of GTE, it was not possible to assign these spectral shifts exclusively to EGCg. Nevertheless, given that EGCg is the predominant catechin in GTE and exhibits strong Zn (II)-chelating activity, these changes are likely to reflect, at least in part, the formation of Zn-EGCg complex [15].

The ^1^H NMR spectra of Zn-GTE provided additional insights. The aromatic hydroxyl region (8.0–9.5 ppm) showed sharper and slightly shifted signals compared to GTE alone, including the appearance of a new peak at 9.06 ppm, possibly corresponding to the OH-5 of EGCg (Figure 1d). These changes are consistent with those observed for the Zn-EGCg complex, suggesting the formation of similar structures within the extract. This enhanced signal sharpness may be due to the reduction of intermolecular bonds induced by ZnSO_4_ addition. Some studies also observed that the ^1^H NMR spectrum of flavonoids became significantly sharper upon divalent metal treatment [40,41]. Deshaies et al. reported that the broad OH signal of polyphenols, caused by proton exchange with other protons in the solvent or solute, becomes sharper upon the addition of cadmium, due to the reduction of intermolecular hydrogen bonding [41]. This assertion supports the idea that various proton-bearing substances, which normally interact with the hydroxyl groups of EGCg in green tea, are replaced by Zn (II) upon its addition. The observed spectral features in this study at least support the possible formation of Zn–polyphenol complexes, most plausibly involving EGCg. However, it remains unclear whether EGCg is deprotonated and bound to Zn (II) in Zn-GTE, as it is in the Zn-EGCg complex.

PXRD analysis was conducted to understand the crystalline structure of Zn-GTE. Zn-GTE exhibited a partially crystalline structure, in contrast to the amorphous nature of native GTE (Figure 1e). The diffraction pattern was distinct from the simple sum of the GTE pattern and ZnSO_4_ 7H_2_O pattern, implying the formation of a new Zn-containing complex phase (Figure S1). Although these findings support structural reorganization upon Zn (II) incorporation, they do not definitively confirm the formation of Zn-EGCg.

In conclusion, the present data support the hypothesis that Zn (II) interacts with EGCg and other constituents in green tea, forming coordination complexes that alter the structural and physicochemical properties of the extract. In a previous study investigating Zn speciation in green tea extracts upon Zn supplementation, it was reported that 85% of the added Zn existed as free Zn ions, while 9% were bound to polyphenols [20]. Although Zn speciation was not directly quantified in the present study, this previous finding provides a relevant reference point for estimating the proportion of free versus complexed Zn in our Zn-GTE sample. The GTE used in this study contained 38.1% EGCg of the principal eight catechins, with other catechins such as (−)-Epigallocatechin (EGC), (−)-Epicatechin (EC), and (−)-Epicatechin gallate (ECg) also present in substantial amounts (Table S1). Although EC and ECg have also been reported to exhibit Zn (II)-chelating activity [42], our chelation assay demonstrated that EGCg had the strongest Zn (II) binding capacity among the four major catechins (Figure 3). Given both its high chelation potency and abundance, EGCg is considered the primary contributor to Zn (II) complexation in GTE under typical extract conditions. This finding reinforces previous structure/activity relationship studies and highlights the importance of considering both molecular potency and relative abundance when evaluating functional interactions in complex botanical matrices.

In addition, it remains unclear whether Zn-EGCg maintains a stable structural state within the tea matrix. Previous studies have suggested that the complexation between EGCg and Zn (II) is influenced by external factors such as temperature and pH [13,43]. These findings imply that Zn-EGCg may exist in an equilibrium state rather than a fixed structure. Since the stability of the complex can affect its bioavailability and functionality, further detailed investigations are necessary to account for these environmental effects and to better understand the behavior of the complex under physiological or food-processing conditions.

### 3.2. Effects of Zn-GTE on Gut Microbial Diversity

To investigate the effects of GTE and Zn-GTE on gut microbiota, 16S rRNA gene sequences from mouse fecal samples were analyzed using the Qiime2 pipeline.

Overall, gut microbial diversity decreased in the WD group compared to the ND group, with particularly pronounced reductions observed in the Chao1 and Faith’s phylogenetic diversity (Faith_pd) indices (Figure 4a,b). The Shannon index also showed a decreasing trend, although it did not reach statistical significance (Figure 4c). Interestingly, the effects of Zn-GTE differed from those of GTE and Zn alone. While GTE or Zn supplementation did not improve the Faith_pd or Shannon indices, respectively, Zn-GTE significantly restored them, suggesting that the Zn-GTE complex exerts distinct effects not attributable to GTE or Zn individually. These results imply an interaction between GTE and Zn (II), leading to enhanced gut microbial diversity. Zn (II) is an essential micronutrient for many bacterial species [31], and Zn (II) deficiency has been linked to decreased gut microbial diversity [32]. An increase in microbial diversity is generally considered beneficial, as it enhances the stability and resilience of the gut microbiota against environmental perturbations that may contribute to disease onset [33]. Therefore, the Zn-GTE complex developed in this study may represent a promising strategy to counteract diet-induced disturbances in gut microbial diversity.

### 3.3. Effects of Zn-GTE on Gut Microbial Composition

PLS-DA successfully distinguished the Zn-GTE group from the GTE and Zn groups based on gut bacterial composition at the genus level, demonstrating good model fit and predictive performance (Figure 5b). Several bacterial taxa, including members of the *f_Rikenellaceae*, *f_Ruminococcaceae*, and *g_Akkermansia*, showed high VIP scores, indicating that their relative abundances substantially differed among the three groups (Figure 5d).

In the LEfSe analysis comparing the GTE and Zn-GTE groups, several bacterial taxa belonging to the *o_Bacteroidales* and *o_Clostridiales*, as well as the *f_Rikenellaceae*, were significantly enriched in the Zn-GTE group (Figure 6e). These taxes overlapped with those exhibiting high VIP scores in the PLS-DA model, reinforcing their relevance in distinguishing the microbial profiles between treatments. Notably, these key bacteria are known to possess short-chain fatty acid (SCFA)-producing capabilities [44,45,46], which are crucial for maintaining intestinal health and metabolic balance. These findings suggest that the formation of Zn (II) complexes with green tea components may enhance the gut microbiota-modulating effects of GTE, potentially improving the intestinal environment more effectively than GTE alone. Previous reports have described Zn (II)-biofortified foods as promising tools to support gut health by promoting bacterial fermentation, given the mineral requirements of gut microbes [47]. For example, Reed et al. demonstrated that Zn (II)-biofortified wheat promoted the growth of SCFA-producing bacteria, including *Clostridiales* and *Ruminococcus* [48]. In this context, Zn-GTE may also function as a novel prebiotic compound, analogous to Zn (II)-biofortified crops.

Although GTE and EGCg intake is generally known to promote the growth of *A. muciniphila* [25,49,50], its abundance was significantly reduced in the Zn-GTE group compared to the GTE group (Figure 6e). This decrease may reflect a context-dependent regulatory effect, possibly influenced by the structural modifications of catechins through Zn (II) complexation. In addition, chronic Zn accumulation reportedly exerts toxic effects on members of the phylum *Verrucomicrobia*—including *A. muciniphila*—through mechanisms such as intermetal competition, mis-metallation of metalloproteins, or redox-related activity [51]. Given that *A. muciniphila* is generally regarded as a beneficial bacterium due to its association with improved metabolic and intestinal health [52], this reduction warrants careful interpretation. Some recent studies have indicated that excessive proliferation of *A. muciniphila* may disrupt the balance between mucin secretion and degradation, potentially damaging the intestinal barrier and exacerbating colitis progression [53]. Further in vitro studies are warranted to clarify how Zn-GTE or Zn-EGCg complex influence the growth and mucin-degrading activity of *A. muciniphila*, as well as their broader physiological implications.

### 3.4. Effects of Zn-GTE on Gut Microbial Metabolic Pathway

According to the predicted gut microbial metabolic pathways based on the MetaCyc database, the Zn-GTE treatment induced functional changes in the gut microbiota that were distinct from those observed with GTE alone, consistent with the differences in microbial composition. The Zn-GTE group showed relatively higher abundances of butyrate production (CENTFERM-PWY and PWY-6590), fatty acid β-oxidation (FAO-PWY), and TCA cycle (PWY-3781 and REDCITCYC) pathways compared to the GTE group (Figure 7c). To ensure the accuracy of metabolic pathway predictions, enrichment analysis was performed using the Kyoto Encyclopedia of Genes and Genomes (KEGG) database pathways that showed both statistically significant differences (*p* < 0.05) and substantial changes (fold change > 2) between the Zn-GTE and GTE groups. In the Zn-GTE group, enrichment of pathways such as “butanoate metabolism” and “fatty acid degradation” was also observed (Figure S2a). These suggest that the produced butyrate may have entered the fatty acid β-oxidation pathway and been catabolized. This interpretation is supported by the observation that actual butyrate levels in the cecum remained unchanged in the Zn-GTE group (Figure 8c). The fatty acid β-oxidation pathway generates acetyl-CoA as its final product, which subsequently enters the TCA cycle for complete catabolism. Zhou et al. reported that green tea polyphenols modulate the gut microbial TCA cycle, leading to reductions in serum glucose and lipid levels [54]. The microbial TCA cycle appears to be associated with host oxidative stress related to lipid metabolism [55,56]. It should be noted, however, that the TCA cycle pathway, which was reduced in the WD group compared to the ND group, was not significantly restored in the Zn-GTE group (Figure S3a–e). Nevertheless, the complexation of Zn (II) and GTE may be considered a potential approach to influence gut microbial function.

Our data showed a significant increase in cecal succinate content in the WD group compared to the ND group. However, treatment with both GTE and Zn-GTE significantly suppressed this elevation, along with a concomitant reduction in the abundance of succinate-producing pathways (ARGDEG-PWY, ORNARGDEG-PWY, and ORNDEG-PWY) (Figure 8d). Serena et al. reported that an increased relative abundance of succinate-producing bacteria, along with a decrease in succinate-consuming bacteria, was associated with obesity and metabolic disorders [57]. From this perspective, Zn-GTE may represent a promising dietary intervention to modulate gut microbiota-derived succinate production. On the other hand, other studies have shown that microbiota-derived succinate can exert beneficial effects, including the improvement of glucose homeostasis [58]. Therefore, further investigations are required to determine whether the Zn-GTE-induced reduction in succinate confers metabolic benefits or poses potential trade-offs for the host.

### 3.5. Effects of Zn-GTE on Obesity-Related Symptoms and Zinc Status in the Host

In the mouse experiments, we assessed the anti-obesity and gut microbiota-modulating effects of GTE and Zn-GTE at doses equivalent to typical beverage consumption. Neither GTE nor Zn-GTE significantly attenuated WD-induced obesity-related symptoms, including weight gain, hepatic fat accumulation, or serum glucose and lipid profiles (Table S3), demonstrating that Zn (II) complexation did not enhance the anti-obesity effects of low-dose GTE in this study. Chung et al. reported that a low dose of GTE at 0.5% (*w*/*w*) did not affect body or liver mass, which is consistent with our results, whereas a high dose of GTE at 1% (*w*/*w*) significantly reduced these parameters in obese mice [59]. These results may indeed elicit additional biological effects within physiologically relevant ranges, warranting further investigation.

Previous reports suggested that green tea naturally enriched in zinc and selenium—accumulated from soil by tea plants—exerted more potent protective effects against high-fat/high-sucrose-induced liver injury than standard green tea, without affecting body weight [60]. While our evaluation focused on obesity-related outcomes, these findings raise the possibility that Zn (II) complexation may enhance other bioactivities of green tea not captured in our current endpoints. For instance, Kagaya et al. demonstrated that Zn (II) complexation improved the hepatoprotective effects of EGCg in isolated rat hepatocytes [61], implying that Zn-catechins interactions may modulate specific functional properties. Further studies are warranted to explore the broader health effects of Zn-GTE beyond anti-obesity activity.

Zn homeostasis is closely linked to metabolic health, with the liver playing a central role in systemic Zn regulation [62,63]. Therefore, we further evaluated the Zn-supplementation capacity of Zn-GTE. Our results demonstrated that Zn-GTE effectively increased hepatic Zn levels, comparable to the effect of pure ZnSO_4_ 7H_2_O supplementation (Figure 9b). Although the inhibitory effects of green tea polyphenols on iron absorption have been widely reported [64,65], few studies have examined their influence on Zn absorption. Kim et al. showed that neither green tea nor EGCg inhibited Zn uptake in Caco-2 cell monolayers [66]. Consistent with these findings, our data indicate that Zn-GTE can serve as a bioavailable Zn source, even in the context of obesity.

### 3.6. Limitations and Future Perspectives

This study has several limitations that should be acknowledged. First, while spectroscopic similarities between Zn-EGCg and Zn-GTE suggest the formation of Zn-EGCg-like complexes in the GTE matrix, definitive structural identification remains challenging. Further structural characterization using techniques such as 2D-NMR, extended X-ray absorption fine structure (EXAFS), or MS-based approaches is needed to confirm the exact coordination state of Zn (II) in the complex matrix. In addition, although qualitative analysis of FT-IR spectra provided insight into the possible interaction between Zn (II) and polyphenols, more detailed interpretation of vibrational modes remains limited. In future studies, applying infrared curve fitting techniques may enhance the resolution of overlapping bands, thereby enabling a more precise evaluation of coordination-related spectral changes in complex matrices such as Zn-GTE.

Second, translating animal data into human physiology has several limitations due to differences in intestinal physiology and gut microbial composition [67]. Notably, 85% of the sequences corresponding to bacterial genera identified in the murine gut are absent from the human microbiome [68]. Further validation in human-based systems is warranted to confirm the observed effects and bioavailability of Zn-polyphenol complexes in vivo.

## 4. Materials and Methods

### 4.1. Chemicals and Reagents

The (−)-epicatechin (EC) (purity ≥ 99%), (−)-epigallocatechin (EGC) (purity ≥ 99%), (−)-epicatechin-3-gallate (ECg) (purity ≥ 99%), (+)-catechin ((+)C) (purity ≥ 99%), and (−)-gallocatechin-3-gallate (GCg) (purity ≥ 98%) were purchased from Nagara Science, Co., Ltd. (Gifu, Japan). The (−)-gallocatechin (GC) (purity ≥ 98%) was purchased from Cayman Chemical Co. (Ann Arbor, MI, USA). The (−)-epigallocatechin-3-gallate (EGCg) (purity ≥ 98%) was purchased from Adipogen Life Sciences, Inc. (San Diego, CA, USA). Lard and cellulose were purchased from Sigma-Aldrich Co., LLC. (Tokyo, Japan). Soybean oil, cholesterol, choline bitartrate, methionine, fructose, and L(+)-ascorbic acid (purity ≥ 99.6%) were purchased from Nacalai Tesque, Inc. (Kyoto, Japan). AIN-93G mineral mix and AIN-93G vitamin mix were purchased from Oriental Yeast Co., Ltd. (Tokyo, Japan). Corn starch was purchased from Sanwa Starch Co., Ltd. (Nara, Japan). Edible Acid Casein 30–60 Mesh was purchased from (Meggle, Wasserburg am Inn., Germany). Sucrose was purchased from Introduction of Hayashi Pure Chemical Ind., Ltd. (Osaka, Japan). FluoZin™-3, Tetrapotassium Salt, cell impermeant was purchased from Thermo Fisher Scientific, Inc. (Waltham, MA, USA).

### 4.2. Sample Preparation

#### 4.2.1. Green Tea Extract (GTE) and Zn-GTE Preparation

Green tea leaves (cv. Yutakamidori) were harvested in Kagoshima Prefecture in Japan. Briefly, 50 g crude tea leaves were brewed with 1 L of hot water at 90 °C for 30 min. The green tea leaves were then immediately separated by centrifugation (3000× *g*, 15 min, 4 °C) and filtration (ADVANTEC, No.131, 600 mm). For preparation of Zn-GTE, 0.382 g of ZnSO_4_ 7H_2_O as solution was added to 700 g fresh supernatant, followed by stirring at 500 rpm for 5 min at 25 °C. The infusions were lyophilized and powdered as GTE or Zn-GTE for subsequent experiments. The principal 8 catechins’ content (EC, EGC, ECg, EGCg, (+)C, GC, Cg, and GCg) of GTE were analyzed by high-performance liquid chromatography (HPLC) as described in our previous study [6].

#### 4.2.2. Zn-EGCg Preparation

EGCg (111.5 µmol) was dissolved in 100 mL of ultrapure water and stirred at 500 rpm at 25 °C. Meanwhile, 111.5 µmol of ZnSO_4_ 7H_2_O (dissolved in 0.714 mL ultrapure water) was added into EGCg solution and reacted for 5 min. The product was lyophilized and powdered as Zn-EGCg for subsequent experiments.

### 4.3. Characterization of Zn-GTE and Zn-EGCg

Zn-GTE and Zn-EGCg were analyzed by different analytical techniques, including UV–Visible spectroscopy (UV–Vis), Fourier transform infrared spectroscopy (FT-IR), ^1^H nuclear magnetic resonance (NMR), and powder X-ray diffraction (PXRD) according to our previous study [69].

#### 4.3.1. UV–Visible Spectroscopy (UV–Vis) Analysis

UV–Vis spectra of samples from 200 to 800 nm (2 nm width pass) were performed in a BioMate 160 spectrophotometer (Thermo Fisher Scientific, Waltham, MA, USA). The concentration of GTE and Zn-GTE was adjusted to 0.01 mg/mL. EGCg and Zn-EGCg were measured by first dissolving EGCg and ZnSO_4_ 7H_2_O in ultrapure water to a final concentration of 320 μM. The EGCg solution was then mixed with either ultrapure water or the ZnSO_4_ 7H_2_O solution at a 1:1 volume ratio, allowed to stand for 5 min, and subsequently diluted 5-fold before measurement. Ultrapure water was used as blank.

#### 4.3.2. Fourier Transform Infrared Spectroscopy (FT-IR) Analysis

Infrared absorption wavelengths were measured via an FT-IR/IRT-3000 ATR-30-Z spectrophotometer (JASCO Corporation, Tokyo, Japan) in the spectral range of 4000–400 cm^−1^. Attenuated total reflection (ATR) measurements were conducted by evenly spreading the powder sample over the entire surface of the ATR crystal. The sample (~5 mg) was then firmly pressed against the prism to ensure good contact during compression.

#### 4.3.3. ^1^H Nuclear Magnetic Resonance (NMR) Analysis

The ^1^H NMR spectra were measured using a nuclear magnetic resonance spectrometer 600 MHz (JEOL-ECA600, JEOL, Tokyo, Japan). The ^1^H NMR measurements were performed in 8 scans (1 min). GTE and Zn-GTE were dissolved in DMSO-d6 and measured (2 mg/0.7 mL). For EGCg and Zn-EGCg measurement, EGCg and ZnSO_4_·7H_2_O were dissolved in DMSO-d_6_ to a final concentration of 320 μM. The EGCg solution was then mixed with either DMSO-d_6_ or the ZnSO_4_·7H_2_O solution at a 1:1 volume ratio, allowed to stand for 5 min, and subsequently subjected to ^1^H NMR measurement.

#### 4.3.4. Powder X-Ray Diffraction (PXRD) Analysis

PXRD patterns were obtained by a PANalytical X’Pert PRO MPD instrument (Malvern Panalytical, Almelo, Netherlands) in the 2θ range of 5 to 40 degrees. The measurement conditions were as follows: divergent slit (DS) size: 0.1089°, sample width: 10.00 mm, measurement temperature: 25 °C, and target: Cu. Approximately 0.1 g of the sample was used.

### 4.4. Measurement of Zn (II) Chelating Ability of GTE and Its Catechins

Zn (II)-specific fluorophore FluoZin-3 was used to evaluate the Zn (II) chelating ability of the samples, based on a previously reported method with slight modifications [15]. Briefly, 50 µL of 3 µM ZnSO_4_·7H_2_O and 50 µL of each test sample (1.0 mg/mL GTE, 0.12 mg/mL EGCg, 0.01 mg/mL ECg, 0.10 mg/mL EGC, and 0.03 mg/mL EC) dissolved in ultrapure water were added to a 96-well microplate and incubated at room temperature for 5 min. Subsequently, 50 µL of 3 µM FluoZin-3 dissolved in 3× PBS was added to each well, and fluorescence was immediately measured at 494 nm (excitation) and 516 nm (emission) using a spectrophotometer (Infinite 200 Pro MPlex, Tecan Ltd., Männedorf, Switzerland). The Zn (II) chelating ability of each sample was calculated as follows:

Zn (II) chelating ability (%) = 100 − {(FI_s_ − FI_sb_)/(FI_ct_ − FI_b_)}

FI_s_: Fluorescence intensity of the sample;
FI_sb_: Fluorescence intensity of the sample blank;
FI_ct_: Fluorescence intensity of the control;
FI_b_: Fluorescence intensity of the control blank.

### 4.5. Animal Experiment Design

All animal experiments were conducted in compliance with the Guidelines of the Animal Care and Use Committee of Kagoshima University and approved by the Animal Ethics Committee of Kagoshima University (Permission No. A20009). Five-week-old C57BL/6N male mice were purchased from Japan SLC Inc. (Shizuoka, Japan) and housed separately in cages with wood shavings bedding and given free access to water and feed. Mice were exposed to a 12-h light/dark cycle and an appropriate temperature (23.5 °C). After acclimatization for 1 week (6 weeks old), the mice were randomly divided into six groups (n = 4, 5): normal diet (ND), Western diet (WD), WD + 0.50% GTE (*wt*/*wt*, GTE), WD + 0.51% Zn-GTE (*wt*/*wt*, Zn-GTE), and WD + 0.01 % ZnSO_4_ 7H_2_O (*wt*/*wt*, Zn) (Table 1). The sample size was set following our previous research to balance statistical robustness with the 3Rs’ “Reduction” guideline in animal studies [70]. The dose of 0.50% GTE in the diet was selected based on body surface area normalization [71], which corresponds to a human equivalent dose of approximately 650 mL/day for a 60 kg adult human—comparable to the typical daily green tea consumption in Japan (~700 mL/day) [72]. Normal water was supplied to the ND group, while 4% sugar water containing 18.9 g/L sucrose and 23.1 g/L fructose was provided to the WD groups. After 12 weeks of test diets, mice were fasted for 12 h and then painlessly anesthetized with isoflurane.

### 4.6. Gut Microbiota Analysis

#### 4.6.1. 16S rRNA Sequencing from Mice Feces

The fresh feces from each mouse were collected at the end of the experiment (18 weeks old) and immediately stored at −80 °C until required. The fecal DNA was extracted by FastDNA SPIN kit for Feces (MP Bio Japan K. K., Tokyo, Japan) according to the manufacturer’s procedure. The V3–V4 region of the 16S rRNA gene was amplified, as described in our previous study [73].

#### 4.6.2. 16S rRNA Sequence Processing

Chimeric and noise sequences were removed with the “DADA2”plugin in Qiime2 (version 2024.10), and representative sequences and final amplicon sequence variables (ASVs) were output. ASVs with 97% similarity to the Greengene database (version 13_8) were used to estimate the phylogeny using the “feature-classifier” plugin. Alpha- and beta-diversities were also assessed with Qiime2 “diversity” plugin. Differential bacterial taxa among the groups were identified as gut microbial biomarkers through linear discriminant analysis (LDA) effect size (LEfSe), with a logarithmic LDA score threshold of 2.0.

#### 4.6.3. Gut Microbial Metabolic Pathway Prediction by PICRUSt2

PICRUSt2 (Phylogenetic Investigation of Communities by Reconstruction of Unobserved States, version 2.5.2) software was applied to predict gut microbial metabolic pathway abundance. Metagenome predictions (PICRUSt2) against Metabolic Pathways From all Domains of Life (MetaCyc) and Kyoto Encyclopedia of Genes and Genomes (KEGG) databases were performed based on the ASVs obtained from 16S rRNA Sequencing.

### 4.7. Cecal Organic Acids Analysis

The cecum contents from each mouse were homogenized in a 10 mM NaOH solution by vortex for 20 s, and then centrifuged at 5800× *g* for 10 min. The obtained supernatants were filtered (Centrifugal Filters Ultracel-10k, Merck Millipore, Billerica, MA, USA) to remove impurities. Organic acids (acetate, propionate, butyrate, succinate, and lactate) in the cecum were measured by a Jasco HPLC system (Tokyo, Japan) using a post-column reaction. The HPLC system consisted of an intelligent sampler (AS-950-10), an intelligent column oven (CO-965), a quaternary gradient pump (PU-2080 Plus), and an ultraviolet detector (UV-2070 Plus). Three Shodex RSpack KC-811 columns (6 µm, 300 mm × 8.0 mm inner diameter) at 60 °C were fitted to the equipment. The injection volume was 20 µL. Each organic acid’s content in the cecum was calculated from the retention time via the standard reference materials and expressed as µmol/g cecum.

### 4.8. Measurement of Serum Biochemical Indexes

Mouse blood was collected in a tube with coagulant (Separable microtubes, Fuchigami Co., Ltd., Kyoto, Japan). After coagulation for 30 min at 24 °C at room temperature, the serum was separated by centrifugation at 1000× *g* for 10 min (tabletop micro cooling centrifuge, Kubota Co., Ltd., Tokyo, Japan). The serum samples were stored at −80 °C until required. Serum levels of aspartate aminotransferase (ALT), alanine aminotransferase (AST), total cholesterol (T-Cho), high-density lipoprotein cholesterol (HDLc), triglyceride (TG), and glucose were measured using an automatic analyzer for clinical chemistry (Spotchem EZ SP-4430, Arkray, Kyoto, Japan). The serum concentration of insulin was measured with an ELISA kit (Wako Pure Chemical Co., Osaka, Japan).

### 4.9. Measurement of Zn Content in Serum and Liver

Serum and hepatic Zn content were measured using the Metalloassay Kit (Metallogenics Co., Chiba, Japan), following the manufacturer’s instructions. Serum samples were measured directly. Liver samples were digested with HNO_3_ at 60 °C for 1 h and then centrifuged at 9100× *g* for 10 min to remove impurities. The pH of the resulting supernatants was subsequently neutralized with 10M NaOH prior to analysis.

### 4.10. Statistical Analysis

Data were expressed as mean ± standard error of the mean. Significant differences between groups were calculated from one-way analysis of variance (ANOVA) followed by Tukey’s test using the R software package “multcomp” (version 1.4.28). Statistical significance was set at *p* < 0.05. Significant differences between sample groups in beta-diversity were determined by pairwise permutational multivariate ANOVA (PERMANOVA) test with 999 permutations using the R software package “pairwiseAdonis” (version 0.4.1). Obtained *p*-values were adjusted by Benjamini–Hochberg correction to avoid Type 1 errors, and the *q*-value < 0.05 was considered significant in beta-diversity analysis. Principal component analysis (PCA) and partial least squares discriminant analysis (PLS-DA) were performed using the R software package “ropls” (version 1.36.0).

## 5. Conclusions

Zinc (II)-complexed green tea extract (Zn-GTE) was prepared by adding Zn (II) to a green tea infusion. The structural analyses using UV–Vis, FT-IR, ^1^H NMR, and PXRD supported the potential formation of Zn-EGCg complex in Zn-GTE. Furthermore, Zn-GTE administration restored alpha-diversity in a WD-induced dysbiosis mouse model at a typical daily consumption. Especially, Zn-GTE enriched members of *o_Clostridiales*, *o_Bacteroidales*, and *f_Rikenellaceae*, while reducing the abundance of *g_Akkermansia*. These findings provide fundamental insight into the interaction between Zn (II) and green tea constituents and suggest its potential to modulate gut microbiota under daily intake conditions.

## Figures and Tables

**Table 1:** Dietary compositions of each group in this study.

Component (%)	ND	WD	GTE	Zn-GTE	Zn
Lard	3	30	30	30	30
Soybean oil	3	3	3	3	3
Corn Starch	45	16.5	16	16.5	16
Casein	20	20	20	20	20
Sucrose	20	20	20	20	20
Cellulose	4	4	4	4	4
Mineral Mix	3.5	3.5	3.5	3.5	3.5
Vitamin Mix	1	1	1	1	1
Cholesterol	0	1.5	1.5	1.5	1.5
Choline Bitartrate	0.2	0.2	0.2	0.2	0.2
Methionine	0.3	0.3	0.3	0.3	0.3
GTE	-	-	0.5	-	-
Zn-GTE	-	-	-	0.51	-
ZnSO_4_ 7H_2_O	-	-	-	-	0.01
Total calories (kcal/100 g)	358.0	511.2	509.4	509.4	511.2
Zn (µg/g)	29.1	29.1	29.4	59.5	59.1

## Data Availability

Data are contained within the article.

## Supplementary Materials

The following supporting information can be downloaded at: https://www.mdpi.com/article/10.3390/molecules30153191/s1, Figure S1. PXRD patterns of Zn-GTE and ZnSO_4_ 7H_2_O. Figure S2. Kyoto Encyclopedia of Genes and Genomes (KEGG) pathway enrichment analysis of upregulated (a) and downregulated (b) pathways in Zn-GTE group compared to GTE group. Figure S3. Effect of samples on gut microbial metabolic pathway detected in volcano plot between GTE and Zn-GTE based on Metabolic Pathways From all Domains of Life (MetaCyc) database. The relative abundance of (a) CENTFERM-PWY, (b) PWY-6590, (c) FAO-PWY, (d) PWY-3781, and (e) REDCITCYC. ND group was set as control value 1. The data represent mean ± SE and different letters in the same column indicate significant differences (*p* < 0.05). Table S1. Each catechin content of GTE. Table S2. The log2 fold change (log2FC) values of Metabolic Pathways From all Domains of Life (MetaCyc) pathways that are significantly different between groups (*p* < 0.05). Table S3. Effect of samples on final body weight, liver weight, hepatic triglyceride (TG) content, and serum biochemical indexes. The numerical values with different letters significantly differ (*p* < 0.05) in the same row.

## Abbreviations

The following abbreviations are used in this manuscript:

EGCg	(−)-Epigallocatechin gallate
ECg	(−)-Epicatechin gallate
EGC	(−)-Epigallocatechin
EC	(−)-Epicatechin
GCg	(−)-Gallocatechin gallate
Cg	(−)-Catechin gallate
GC	(−)-Gallocatechin
C	(+)-Catechin
GTE	Green tea extract
PCA	Principal component analysis
PLS-DA	Partial least squares discriminant analysis
ND	Normal diet
HFD	High-fat diet
WD	Western diet
UV–Vis	UV–Visible spectroscopy
FT-IR	Fourier transform infrared spectroscopy
NMR	Nuclear magnetic resonance
PXRD	Powder X-ray diffraction
LEfSe	Linear Discriminant Analysis LDA Effect Size
PICRUSt2	Phylogenetic Investigation of Communities by Reconstruction of Unobserved States
MetaCyc	Metabolic Pathways From all Domains of Life
KEGG	Kyoto Encyclopedia of Genes and Genomes
ANOVA	Analysis of variance
PERMANOVA	Permutational multivariate analysis of variance

## References

[1] Zhao T., Li C., Wang S., Song X. (2022). Green Tea (*Camellia sinensis*): A Review of Its Phytochemistry, Pharmacology, and Toxicology. Molecules.

[2] Liu Z., Chen Q., Zhang C., Ni L. (2022). Comparative Study of the Anti-Obesity and Gut Microbiota Modulation Effects of Green Tea Phenolics and Their Oxidation Products in High-Fat-Induced Obese Mice. Food Chem..

[3] Oh J.W., Muthu M., Pushparaj S.S.C., Gopal J. (2023). Anti-cancer Therapeutic Effects of Green Tea Catechins (GTCs) When Integrated with Antioxidant Natural Components. Molecules.

[4] Huang Z., Zhang L., Xuan J., Yang L., Zhao T., Peng W. (2024). Tea for Histamine Anti-Allergy: Component Analysis of Tea Extracts and Potential Mechanism for Treating Histamine Anti-Allergy. Front. Pharmacol..

[5] Shen J., Xie J., Ye L., Mao J., Sun S., Chen W., Wei S., Ruan S., Wang L., Hu H. (2024). Neuroprotective Effect of Green Tea Extract (-)-Epigallocatechin-3-Gallate in a Preformed Fibril-Induced Mouse Model of Parkinson’s Disease. Neuroreport.

[6] Orita T., Chogahara S., Okuda M., Sakao K., Miyata T., Hou D.X. (2023). Extraction Efficiency and Alpha-Glucosidase Inhibitory Activities of Green Tea Catechins by Different Infusion Methods. Foods.

[7] Onakpoya I., Spencer E., Heneghan C., Thompson M. (2014). The Effect of Green Tea on Blood Pressure and Lipid Profile: A Systematic Review and Meta-Analysis of Randomized Clinical Trials. Nutr. Metab. Cardiovasc. Dis..

[8] Li Z., Kang M., Zhang S., Zhang S., Dongye Z., Wang L., Chen C., Cheng X., Qian Y., Ren Y. (2023). The Improved Inhibition of Mn (II)-EGCG on α-Glucosidase: Characteristics and Interactions Properties. J. Mol. Struct..

[9] Alhafez M., Kheder F., Aljoubbeh M. (2022). Synthesis, Antioxidant Activity and Antibacterial Study of EGCG Complexes with Iron(III) Ions. Results Chem..

[10] Farhan M., Khan H.Y., Oves M., Al-Harrasi A., Rehmani N., Arif H., Hadi S.M., Ahmad A. (2016). Cancer Therapy by Catechins Involves Redox Cycling of Copper Ions and Generation of Reactive Oxygenspecies. Toxins.

[11] Wang X., Feng Y., Chen C., Yang H., Yang X. (2020). Preparation, Characterization and Activity of Tea Polyphenols-Zinc Complex. LWT.

[12] Zhang L.C., Yu H.N., Sun S.L., Yang J.G., He G.Q., Ruan H., Shen S.R. (2008). Investigations of the Cytotoxicity of Epigallocatechin-3-Gallate against PC-3 Cells in the Presence of Cd2+ in Vitro. Toxicol. In Vitro.

[13] Gokhale K.M., Yadav V.D. (2020). Study on Synthesis, in-Vitro Anti-Inflammatory and Anti-cancer Activity of L-Catechin Metal Complexes. Eur. J. Mol. Clin. Med..

[14] Dabbagh-Bazarbachi H., Clergeaud G., Quesada I.M., Ortiz M., O’Sullivan C.K., Fernández-Larrea J.B. (2014). Zinc Ionophore Activity of Quercetin and Epigallocatechin-Gallate: From Hepa 1-6 Cells to a Liposome Model. J. Agric. Food Chem..

[15] Clergeaud G., Dabbagh-Bazarbachi H., Ortiz M., Fernández-Larrea J.B., O’Sullivan C.K. (2016). A Simple Liposome Assay for the Screening of Zinc Ionophore Activity of Polyphenols. Food Chem..

[16] Sun S.L., He G.Q., Yu H.N., Yang J.G., Borthakur D., Zhang L.C., Shen S.R., Das U.N. (2008). Free Zn2+ Enhances Inhibitory Effects of EGCG on the Growth of PC-3 Cells. Mol. Nutr. Food Res..

[17] Chen X., Yu H., Shen S., Yin J. (2007). Role of Zn2+ in Epigallocatechin Gallate Affecting the Growth of PC-3 Cells. J. Trace Res. Elem. Med. Biol..

[18] Zhang H., Zhao Y. (2015). Preparation, Characterization and Evaluation of Tea Polyphenol-Zn Complex Loaded β-Chitosan Nanoparticles. Food Hydrocoll..

[19] Kader D.A., Aziz D.M., Mohammed S.J., Maarof N.N.N., Karim W.O., Mhamad S.A., Rashid R.M., Ayoob M.M., Kayani K.F., Qurbani K. (2024). Green Synthesis of ZnO/Catechin Nanocomposite: Comprehensive Characterization, Optical Study, Computational Analysis, Biological Applications and Molecular Docking. Mater. Chem. Phys..

[20] Pohl P., Prusisz B. (2007). Fractionation Analysis of Manganese and Zinc in Tea Infusions by Two-Column Solid Phase Extraction and Flame Atomic Absorption Spectrometry. Food Chem..

[21] Dube A., Nicolazzo J.A., Larson I. (2010). Chitosan Nanoparticles Enhance the Intestinal Absorption of the Green Tea Catechins (+)-Catechin and (-)-Epigallocatechin Gallate. Eur. J. Pharm. Sci..

[22] Clifford M.N., Van Der Hooft J.J.J., Crozier A. (2013). Human Studies on the Absorption, Distribution, Metabolism, and Excretion of Tea Polyphenols1-3. Am. J. Clin. Nutr..

[23] Hussain K., Yang Y., Wang J., Bian H., Lei X., Chen J., Li Q., Wang L., Zhong Q., Fang X. (2022). Comparative Study on the Weight Loss and Lipid Metabolism by Tea Polyphenols in Diet Induced Obese C57BL/6J Pseudo Germ Free and Conventionalized Mice. Food Sci. Hum. Wellness.

[24] Yuan X., Long Y., Ji Z., Gao J., Fu T., Yan M., Zhang L., Su H., Zhang W., Wen X. (2018). Green Tea Liquid Consumption Alters the Human Intestinal and Oral Microbiome. Mol. Nutr. Food Res..

[25] Liu X., Zhao K., Jing N., Zhao Y., Yang X. (2020). EGCG Regulates Fatty Acid Metabolism of High-Fat Diet-Fed Mice in Association with Enrichment of Gut Akkermansia Muciniphila. J. Funct. Foods.

[26] Song Q., Zhang X. (2022). The Role of Gut–Liver Axis in Gut Microbiome Dysbiosis Associated NAFLD and NAFLD-HCC. Biomedicines.

[27] Canale M.P., Noce A., Di Lauro M., Marrone G., Cantelmo M., Cardillo C., Federici M., Di Daniele N., Tesauro M. (2021). Gut Dysbiosis and Western Diet in the Pathogenesis of Essential Arterial Hypertension: A Narrative Review. Nutrients.

[28] Malesza I.J., Malesza M., Walkowiak J., Mussin N., Walkowiak D., Aringazina R., Bartkowiak-Wieczorek J., Mądry E. (2021). High-Fat, Western-Style Diet, Systemic Inflammation, and Gut Microbiota: A Narrative Review. Cells.

[29] Suárez-Zamorano N., Fabbiano S., Chevalier C., Stojanović O., Colin D.J., Stevanović A., Veyrat-Durebex C., Tarallo V., Rigo D., Germain S. (2015). Microbiota Depletion Promotes Browning of White Adipose Tissue and Reduces Obesity. Nat. Med..

[30] Hrncir T. (2022). Gut Microbiota Dysbiosis: Triggers, Consequences, Diagnostic and Therapeutic Options. Microorganisms.

[31] Molinari R., Merendino N., Costantini L. (2022). Polyphenols as Modulators of Pre-Established Gut Microbiota Dysbiosis: State-of-the-Art. BioFactors.

[32] Le Chatelier E., Nielsen T., Qin J., Prifti E., Hildebrand F., Falony G., Almeida M., Arumugam M., Batto J.M., Kennedy S. (2013). Richness of Human Gut Microbiome Correlates with Metabolic Markers. Nature.

[33] Clemente-Suárez V.J., Beltrán-Velasco A.I., Redondo-Flórez L., Martín-Rodríguez A., Tornero-Aguilera J.F. (2023). Global Impacts of Western Diet and Its Effects on Metabolism and Health: A Narrative Review. Nutrients.

[34] Nakano H., Wu S., Sakao K., Hara T., He J., Garcia S., Shetty K., Hou D.X. (2020). Bilberry Anthocyanins Ameliorate NAFLD by Improving Dyslipidemia and Gut Microbiome Dysbiosis. Nutrients.

[35] Huang Y., Lu F., Guo Y., Cheng K.K., Wang Z., Li W., Dong J., Huang B., Cui X. (2025). Solvent Selection in the Quality Analysis of White Tea by Nuclear Magnetic Resonance Spectroscopy. LWT.

[36] Yasarawan N., Thipyapong K., Sirichai S., Ruangpornvisuti V. (2013). Fundamental Insights into Conformational Stability and Orbital Interactions of Antioxidant (+)-Catechin Species and Complexation of (+)-Catechin with Zinc(II) and Oxovanadium(IV). J. Mol. Struct..

[37] Diniz P.H.G.D., Barbosa M.F., De Melo Milanez K.D.T., Pistonesi M.F., De Araújo M.C.U. (2016). Using UV-Vis Spectroscopy for Simultaneous Geographical and Varietal Classification of Tea Infusions Simulating a Home-Made Tea Cup. Food Chem..

[38] López-Martínez L., López-de-Alba P.L., García-Campos R., De León-Rodríguez L.M. (2003). Simultaneous Determination of Methylxanthines in Coffees and Teas by UV-Vis Spectrophotometry and Partial Least Squares. Anal. Chim. Acta.

[39] Yousefbeyk F., Ebrahimi-Najafabadi H., Dabirian S., Salimi S., Baniardalani F., Moghadam F.A., Ghasemi S. (2023). Phytochemical Analysis and Antioxidant Activity of Eight Cultivars of Tea (*Camellia sinensis*) and Rapid Discrimination with FTIR Spectroscopy and Pattern Recognition Techniques. Pharm. Sci..

[40] Primikyri A., Mazzone G., Lekka C., Tzakos A.G., Russo N., Gerothanassis I.P. (2015). Understanding Zinc(II) Chelation with Quercetin and Luteolin: A Combined NMR and Theoretical Study. J. Phys. Chem. B.

[41] Deshaies S., Le Guernevé C., Suc L., Mouls L., Garcia F., Saucier C. (2021). Unambiguous Nmr Structural Determination of (+)-Catechin—Laccase Dimeric Reaction Products as Potential Markers of Grape and Wine Oxidation. Molecules.

[42] Quesada I.M., Bustos M., Blay M., Pujadas G., Ardèvol A., Salvadó M.J., Bladé C., Arola L., Fernández-Larrea J. (2011). Dietary Catechins and Procyanidins Modulate Zinc Homeostasis in Human HepG2 Cells. J. Nutr. Biochem..

[43] Zhang L., Liu R., Gung B.W., Tindall S., Gonzalez J.M., Halvorson J.J., Hagerman A.E. (2016). Polyphenol-Aluminum Complex Formation: Implications for Aluminum Tolerance in Plants. J. Agric. Food Chem..

[44] Sun X., Liu Y., Jiang P., Song S., Ai C. (2021). Interaction of Sulfated Polysaccharides with Intestinal Bacteroidales Plays an Important Role in Its Biological Activities. Int. J. Biol. Macromol..

[45] Tavella T., Rampelli S., Guidarelli G., Bazzocchi A., Gasperini C., Pujos-Guillot E., Comte B., Barone M., Biagi E., Candela M. (2021). Elevated Gut Microbiome Abundance of Christensenellaceae, Porphyromonadaceae and Rikenellaceae Is Associated with Reduced Visceral Adipose Tissue and Healthier Metabolic Profile in Italian Elderly. Gut Microbes.

[46] Dong M., Liang X., Zhu T., Xu T., Xie L., Feng Y. (2024). Reoxygenation Mitigates Intermittent Hypoxia-Induced Systemic Inflammation and Gut Microbiota Dysbiosis in High-Fat Diet-Induced Obese Rats. Nat. Sci. Sleep.

[47] Gomes M.J.C., Martino H.S.D., Tako E. (2021). Effects of Iron and Zinc Biofortified Foods on Gut Microbiota in Vivo (*Gallus gallus*): A Systematic Review. Nutrients.

[48] Reed S., Knez M., Uzan A., Stangoulis J.C., Glahn R.P., Koren O., Tako E. (2018). Alterations in the Gut (*Gallus gallus*) Microbiota Following the Consumption of Zinc Biofortified Wheat (*Triticum aestivum*)-Based Diet. J. Agric. Food Chem..

[49] Jeong H.W., Kim J.K., Kim A.Y., Cho D., Lee J.H., Choi J.K., Park M., Kim W. (2020). Tea Encourages Growth of Akkermansia Muciniphila. J. Med. Food.

[50] Xia Y., Zhang X., Jiang M., Zhang H., Wang Y., Zhang Y., Seviour R., Kong Y. (2021). In Vitro Co-Metabolism of Epigallocatechin-3gallate (EGCG) by the Mucin-Degrading Bacterium Akkermansia Muciniphila. PLoS ONE.

[51] Chen L., Wang Z., Wang P., Yu X., Ding H., Wang Z., Feng J. (2021). Effect of Long-Term and Short-Term Imbalanced Zn Manipulation on Gut Microbiota and Screening for Microbial Markers Sensitive to Zinc Status. Microbiol. Spectr..

[52] Rodrigues V.F., Elias-Oliveira J., Pereira Í.S., Pereira J.A., Barbosa S.C., Machado M.S.G., Carlos D. (2022). Akkermansia Muciniphila and Gut Immune System: A Good Friendship That Attenuates Inflammatory Bowel Disease, Obesity, and Diabetes. Front. Immunol..

[53] Qu S., Zheng Y., Huang Y., Feng Y., Xu K., Zhang W., Wang Y., Nie K., Qin M. (2023). Excessive Consumption of Mucin by Over-Colonized Akkermansia Muciniphila Promotes Intestinal Barrier Damage during Malignant Intestinal Environment. Front. Microbiol..

[54] Zhou J., Tang L., Shen C.L., Wang J.S. (2020). Green Tea Polyphenols Boost Gut-Microbiota-Dependent Mitochondrial TCA and Urea Cycles in Sprague–Dawley Rats. J. Nutr. Biochem..

[55] Holmes E., Li J.V., Marchesi J.R., Nicholson J.K. (2012). Gut Microbiota Composition and Activity in Relation to Host Metabolic Phenotype and Disease Risk. Cell Metab..

[56] Cani P.D., Delzenne N.M. (2009). The Role of the Gut Microbiota in Energy Metabolism and Metabolic Disease. Curr. Pharm. Des..

[57] Serena C., Ceperuelo-Mallafré V., Keiran N., Queipo-Ortuño M.I., Bernal R., Gomez-Huelgas R., Urpi-Sarda M., Sabater M., Pérez-Brocal V., Andrés-Lacueva C. (2018). Elevated Circulating Levels of Succinate in Human Obesity Are Linked to Specific Gut Microbiota. ISME J..

[58] De Vadder F., Kovatcheva-Datchary P., Zitoun C., Duchampt A., Bäckhed F., Mithieux G. (2016). Microbiota-Produced Succinate Improves Glucose Homeostasis via Intestinal Gluconeogenesis. Cell Metab..

[59] Chung M.Y., Park H.J., Manautou J.E., Koo S.I., Bruno R.S. (2012). Green Tea Extract Protects against Nonalcoholic Steatohepatitis in Ob/Ob Mice by Decreasing Oxidative and Nitrative Stress Responses Induced by Proinflammatory Enzymes. J. Nutr. Biochem..

[60] Yu J., Yang J., Li M., Yang X., Wang P., Xu J. (2018). Protective Effects of Chinese Fenggang Zinc Selenium Tea on Metabolic Syndrome in High-Sucrose-High-Fat Diet-Induced Obese Rats. Sci. Rep..

[61] Kagaya N., Kawase M., Maeda H., Tagawa Y.-I., Nagashima H., Ohmori H., Yagi K. (2002). Enhancing Effect of Zinc on Hepatoprotectivity of Epigallocatechin Gallate in Isolated Rat Hepatocytes. Biol. Pharm. Bull..

[62] Fathi M., Alavinejad P., Haidari Z., Amani R. (2020). The Effect of Zinc Supplementation on Steatosis Severity and Liver Function Enzymes in Overweight/Obese Patients with Mild to Moderate Non-Alcoholic Fatty Liver Following Calorie-Restricted Diet: A Double-Blind, Randomized Placebo-Controlled Trial. Biol. Trace Elem. Res..

[63] Prasad A.S. (1983). The Role of Zinc in Gastrointestinal and Liver Disease. Clin. Gastroenterol..

[64] Samman S., Sandström B., Toft M.B., Bukhave K., Jensen M., Sørensen S.S., Hansen M. (2001). Green Tea or Rosemary Extract Added to Foods Reduces-Iron Absorption. Am. J. Clin. Nutr..

[65] Ma Q., Kim E.Y., Lindsay E.A., Han O. (2011). Bioactive Dietary Polyphenols Inhibit Heme Iron Absorption in a Dose-Dependent Manner in Human Intestinal Caco-2 Cells. J. Food Sci..

[66] Kim E.Y., Pai T.K., Han O. (2011). Effect of Bioactive Dietary Polyphenols on Zinc Transport across the Intestinal Caco-2 Cell Monolayers. J. Agric. Food Chem..

[67] Hugenholtz F., de Vos W.M. (2018). Mouse Models for Human Intestinal Microbiota Research: A Critical Evaluation. Cell. Mol. Life Sci..

[68] Ley R.E., Bä Ckhed F., Turnbaugh P., Lozupone C.A., Knight R.D., Gordon J.I. (2005). Obesity Alters Gut Microbial Ecology. Proc. Natl. Acad. Sci. USA.

[69] Nakamura M., Urakawa D., He Z., Akagi I., Hou D.X., Sakao K. (2023). Apoptosis Induction in HepG2 and HCT116 Cells by a Novel Quercetin-Zinc (II) Complex: Enhanced Absorption of Quercetin and Zinc (II). Int. J. Mol. Sci..

[70] Xie K., He X., Chen K., Sakao K., Hou D.X. (2020). Ameliorative Effects and Molecular Mechanisms of Vine Tea on Western Diet-Induced NAFLD. Food Funct..

[71] Reagan-Shaw S., Nihal M., Ahmad N. (2008). Dose Translation from Animal to Human Studies Revisited. FASEB J..

[72] Sakata R., Nakamura T., Torimura T., Ueno T., Sata M. (2013). Green Tea with High-Density Catechins Improves Liver Function and Fat Infiltration in Non-Alcoholic Fatty Liver Disease (NAFLD) Patients: A Double-Blind Placebo-Controlled Study. Int. J. Mol. Med..

[73] Wu S., Hu R., Nakano H., Chen K., Liu M., He X., Zhang H., He J., Hou D.X. (2018). Modulation of Gut Microbiota by Lonicera Caerulea l. Berry Polyphenols in a Mouse Model of Fatty Liver Induced by High Fat Diet. Molecules.

