# Supplementary material for: Creation of Zinc (II)-Complexed Green Tea and Its Effects on Gut Microbiota by Daily Green Tea Consumption
Source: Molecules. 2025 Jul 30;30(15):3191. doi: 10.3390/molecules30153191 (PMC12348637; doi:10.3390/molecules30153191)
Supplement: Supplementary file 1 [file molecules-30-03191-s001.zip › molecules-3753597-supplementary.pdf]

molecules

Supplementary material

Creation of Zinc (II)-Complexed Green Tea and Its Effects on Gut Microbiota by Daily Green Tea Consumption

Tsukasa Orita [1], Daichi Ijiri [1,2], De-Xing Hou [1,3] and Kozue Sakao [1,3,*]

[1] The United Graduate School of Agricultural Sciences, Kagoshima University, Kagoshima 890-0065, Japan
[2] Joint Faculty of Veterinary Medicine, Kagoshima University, Kagoshima 890-0065, Japan
[3] Faculty of Agriculture, Kagoshima University, Kagoshima 890-0065, Japan
* Correspondence: k0939002@kadai.jp; Tel.: +81-99-285-8650

Table S1. Each catechin content of GTE.

	g/100 g GTE
EC	3.49
EGC	10.24
ECg	1.48
EGCg	12.47
(+)C	0.70
GC	2.58
Cg	0.41
GCg	1.40
Gallate catechins (ECg+EGCg+Cg+GCg)	15.78
Free catechins (EC+EGC+C+GC)	17.00
Total catechins	32.78

Table S2. The log2 fold change (log2FC) values of Metabolic Pathways From all Domains of Life (MetaCyc) pathways that are significantly different between groups (p < 0.05).

**Pairs**		**Entry**	**Function**	**log2FC**
WD/ND	Sig_up	P261-PWY	coenzyme M biosynthesis I	4.18
		GOLPDLCAT-PWY	superpathway of glycerol degradation to 1,3-propanediol	3.74
		PWY-5677	succinate fermentation to butanoate	2.60
		P163-PWY	L-lysine fermentation to acetate and butanoate	2.42
		PWY-6628	superpathway of L-phenylalanine biosynthesis	2.21
		PWY-6630	superpathway of L-tyrosine biosynthesis	2.19
		PWY-1541	superpathway of taurine degradation	1.78
		PWY-7003	glycerol degradation to butanol	1.74
		PWY-6749	CMP-legionaminate biosynthesis I	1.33
		PWY-7090	UDP-2,3-diacetamido-2,3-dideoxy-α-D-mannuronate biosynthesis	1.13
		PWY-7332	superpathway of UDP-N-acetylglucosamine-derived O-antigen building blocks biosynthesis	1.03
	Sig_down	PWY-7315	dTDP-N-acetylthomosamine biosynthesis	−1.10
		PWY-3781	aerobic respiration I (cytochrome c)	−1.29
		PWY-5920	heme biosynthesis	−1.35
		P23-PWY	reductive TCA cycle I	−1.54
		TYRFUMCAT-PWY	tyrosine degradation	−1.87
		PWY-6876	isopropanol biosynthesis (engineered)	−4.11
GTE/WD	Sig_up	P261-PWY	coenzyme M biosynthesis I	1.74
	Sig_down	RUMP-PWY	formaldehyde oxidation I	−1.24
		HEMESYN2-PWY	heme b biosynthesis II (anaerobic)	−1.25
		PWY-7237	myo-, chiro- and scyllo-inositol degradation	−1.26
		PWY-1861	formaldehyde assimilation II (assimilatory RuMP Cycle)	−1.30
		PWY-6630	superpathway of L-tyrosine biosynthesis	−1.52
		PWY-6628	superpathway of L-phenylalanine biosynthesis	−1.54
		PWY0-1261	anhydromuropeptides recycling I	−1.72
		PWY-5177	glutaryl-CoA degradation	−1.75
		PWY-7234	inosine-5'-phosphate biosynthesis III	−1.92
		PWY-5920	superpathway of b heme biosynthesis from glycine	−2.59
		PWY-1541	superpathway of taurine degradation	−2.83
		P105-PWY	TCA cycle IV (2-oxoglutarate decarboxylase)	−3.37
		GOLPDLCAT-PWY	superpathway of glycerol degradation to 1,3-propanediol	−4.56
Zn-GTE/GTE	Sig_up	TYRFUMCAT-PWY	L-tyrosine degradation I	3.14
		PWY-5920	superpathway of heme b biosynthesis from glycine	2.29
		REDCITCYC	TCA cycle VI (Helicobacter)	1.58
		PWY-3781	aerobic respiration I (cytochrome c)	1.41
		FAO-PWY	fatty acid β-oxidation I (generic)	1.40
		CENTFERM-PWY	pyruvate fermentation to butanoate	1.36
		PWY-5177	glutaryl-CoA degradation	1.34
		PWY-6590	superpathway of Clostridium acetobutylicum acidogenic fermentation	1.34

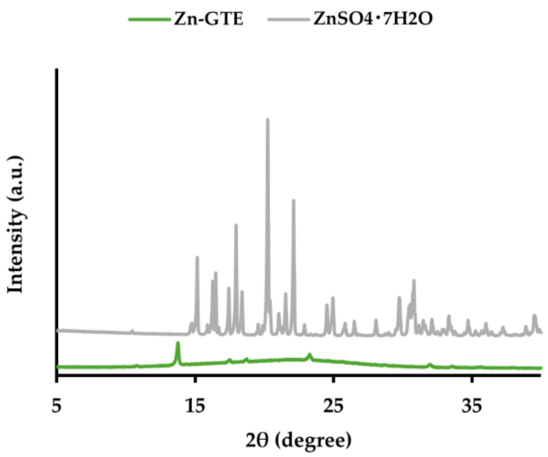

Figure S1. PXRD patterns of Zn-GTE and $ZnSO_4$ $7H_2O$.

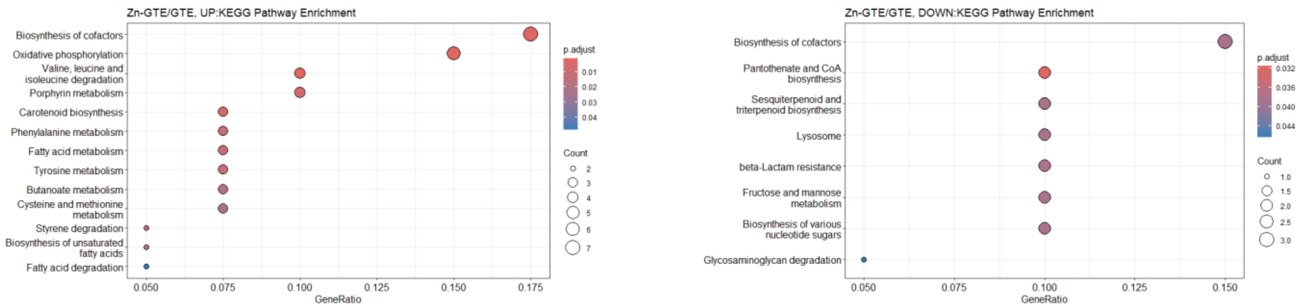

(**a**) (**b**)

Figure S2. Kyoto Encyclopedia of Genes and Genomes (KEGG) pathway enrichment analysis of upregulated (**a**) and downregulated (**b**) pathways in Zn-GTE group compared to GTE group.

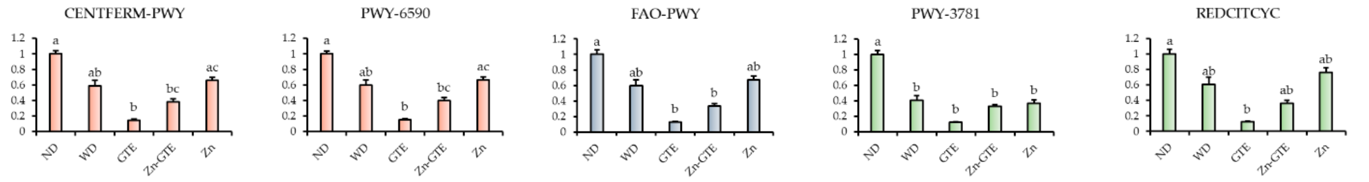

(**a**) (**b**) (**c**) (**d**) (**e**)

Figure S3. Effect of samples on gut microbial metabolic pathway detected in volcano plot between GTE and Zn-GTE based on Metabolic Pathways From all Domains of Life (MetaCyc) database. The relative abundance of (**a**) CENTFERM-PWY, (**b**) PWY-6590, (**c**) FAO-PWY, (**d**) PWY-3781, and (**e**) REDCITCYC. ND group was set as control value 1. The data represent mean ± SE and different letters in the same column indicate significant differences ($p<0.05$).

Table S3. Effect of samples on final body weight, liver weight, hepatic triglyceride (TG) content, and serum biochemical indexes. The numerical values with different letters significantly differ ($p < 0.05$) in the same row.

	ND	WD	GTE	Zn-GTE	Zn
Final body weight (g)	39.3±0.69a	46.1±1.29b	46.2±1.80b	43.0±1.36ab	46.1±0.80b
Liver weight (g)	1.48±0.12a	2.39±0.18a	2.35±0.29a	2.05±0.30a	2.00±0.18a
Hepatic TG (mg/g liver)	155.1±11.8a	292.9±4.9b	306.3±5.9bb	300.5±10.6b	250.4±9.5b
AST (IU/L)	309.0±26.6a	218.2±18ab	175.8±7.3b	211.8±23.4ab	229±33.8ab
ALT (IU/L)	147.8±35.1a	173.2±23.3a	151.8±32.3a	152.0±48.6a	184.0±44.8a
ALT/AST	0.5±0.1a	0.8±0.1a	0.9±0.2a	0.7±0.2a	0.8±0.1a
Total cholesterol (mg/dL)	180.4±18.2a	276.0±14.3ab	281.5±35.8ab	283.8±21.6b	243.5±8.4ab
HDL-Cholesterol (mg/dL)	85.8±6.4a	153.0±6.5b	165.3±14.1b	150.0±8.3b	137.8±3.8b

Non-HDL-Cholesterol (mg/dL)	94.6±12.6a	123.0±9.5a	116.3±26.5a	133.8±14.3a	105.8±5.6a
Triglyceride (mg/dL)	91.4±6a	76.2±4.3a	71.5±4.3a	75.8±8.4a	63.3±4a
Glucose (mg/dL)	169.0±12.2ab	146.0±9.4b	216.8±14.7a	160.0±10ab	131.5±9.8b
Insulin (ng/mL)	2.5±0.4a	3.2±1.5a	3±1.2a	1.3±0.6a	2.0±1.2a
HOMA-IR	1.1±0.3a	1.2±0.6a	1.5±0.4a	0.6±0.3a	0.6±0.3a
